# Second-Tier Next Generation Sequencing Integrated in Nationwide Newborn Screening Provides Rapid Molecular Diagnostics of Severe Combined Immunodeficiency

**DOI:** 10.3389/fimmu.2020.01417

**Published:** 2020-07-09

**Authors:** Janne Strand, Kiran Aftab Gul, Hans Christian Erichsen, Emma Lundman, Mona C. Berge, Anette K. Trømborg, Linda K. Sørgjerd, Mari Ytre-Arne, Silje Hogner, Ruth Halsne, Hege Junita Gaup, Liv T. Osnes, Grete A. B. Kro, Hanne S. Sorte, Lars Mørkrid, Alexander D. Rowe, Trine Tangeraas, Jens V. Jørgensen, Charlotte Alme, Trude E. H. Bjørndalen, Arild E. Rønnestad, Astri M. Lang, Terje Rootwelt, Jochen Buechner, Torstein Øverland, Tore G. Abrahamsen, Rolf D. Pettersen, Asbjørg Stray-Pedersen

**Affiliations:** ^1^Norwegian National Unit for Newborn Screening, Division of Paediatric and Adolescent Medicine, Oslo University Hospital, Oslo, Norway; ^2^Paediatric Research Institute, Division of Paediatric and Adolescent Medicine, Oslo University Hospital, Oslo, Norway; ^3^Department of Paediatrics, Division of Paediatric and Adolescent Medicine, Oslo University Hospital, Oslo, Norway; ^4^Division of Paediatric and Adolescent Medicine, Institute of Clinical Medicine, University of Oslo, Oslo, Norway; ^5^Department of Forensic Biology, Oslo University Hospital, Oslo, Norway; ^6^Department of Immunology and Transfusion Medicine, Oslo University Hospital, Oslo, Norway; ^7^Department of Microbiology, Oslo University Hospital, Oslo, Norway; ^8^Department of Medical Genetics, Oslo University Hospital, Oslo, Norway; ^9^Department of Medical Biochemistry, Oslo University Hospital, Oslo, Norway; ^10^Department of Paediatric Haematology, Division of Paediatric and Adolescent Medicine, Oslo University Hospital, Oslo, Norway; ^11^Department of Obstetrics and Gynaecology, Oslo University Hospital, Oslo, Norway

**Keywords:** SCID - severe combined immunodeficiency, newborn SCID screening, NGS - next generation sequencing, severe T-cell immunodeficiency, TREC analysis

## Abstract

Severe combined immunodeficiency (SCID) and other T cell lymphopenias can be detected during newborn screening (NBS) by measuring T cell receptor excision circles (TRECs) in dried blood spot (DBS) DNA. Second tier next generation sequencing (NGS) with an amplicon based targeted gene panel using the same DBS DNA was introduced as part of our prospective pilot research project in 2015. With *written* parental consent, 21 000 newborns were TREC-tested in the pilot. Three newborns were identified with SCID, and disease-causing variants in *IL2RG, RAG2*, and *RMRP* were confirmed by NGS on the initial DBS DNA. The molecular findings directed follow-up and therapy: the *IL2RG*-SCID underwent early hematopoietic stem cell transplantation (HSCT) without any complications; the leaky *RAG2-*SCID received prophylactic antibiotics, antifungals, and immunoglobulin infusions, and underwent HSCT at 1 year of age. The child with *RMRP*-SCID had complete Hirschsprung disease and died at 1 month of age. Since January 2018, all newborns in Norway have been offered NBS for SCID using 1st tier TRECs and 2nd tier gene panel NGS on DBS DNA. During the first 20 months of nationwide SCID screening an additional 88 000 newborns were TREC tested, and four new SCID cases were identified. Disease-causing variants in *DCLRE1C, JAK3, NBN*, and *IL2RG* were molecularly confirmed on day 8, 15, 8 and 6, respectively after birth, using the initial NBS blood spot. Targeted gene panel NGS integrated into the NBS algorithm rapidly delineated the specific molecular diagnoses and provided information useful for management, targeted therapy and follow-up i.e., X rays and CT scans were avoided in the radiosensitive SCID. Second tier targeted NGS on the same DBS DNA as the TREC test provided instant confirmation or exclusion of SCID, and made it possible to use a less stringent TREC cut-off value. This allowed for the detection of leaky SCIDs, and simultaneously reduced the number of control samples, recalls and false positives. Mothers were instructed to stop breastfeeding until maternal *cytomegalovirus* (CMV) status was determined. Our limited data suggest that shorter time-interval from birth to intervention, may prevent breast milk transmitted CMV infection in classical SCID.

## Introduction

Early diagnosis is important in severe combined immunodeficiency (SCID). Targeted treatment, molecularly adjusted preconditioning, as well as avoiding infections such as *cytomegalovirus* (CMV) before undergoing hematopoietic stem cell transplantation (HSCT), improves overall prognosis (1–4). Newborn screening (NBS) for SCID was first introduced in the United States in 2008^1^. SCID became part of the US core recommended uniform screening panel (RUSP) in 2010, and since December 2018 all US states have implemented SCID screening as part of their NBS program^2^. Nationwide SCID screening was implemented in Taiwan 2012 (5, 6), and Israel Oct 2015 (6, 7). In Europe, universal SCID screening started in the region of Catalonia in Spain (Jan 2017) (8), Iceland (2017), Switzerland (Jan 2019), Sweden and Germany (Aug 2019) proceeded by pilots (9–11)^3^, and Denmark (Feb 2020) (12). And hospital pilots have been performed (13), or are ongoing in European countries and regions such as France (14), Finland, Poland, Italy, and the Netherlands (15, 16).

SCID and other T cell lymphopenias can be identified during newborn screening (NBS) by measuring T cell receptor excision circles (TRECs) in DBS DNA (17)^4^. Common SCID screening algorithms include quantification of TRECs as 1st tier, sometimes followed by a repeated control blood sample from the baby (18). Based on each laboratory's cut-off values for TRECs, 1–10 per 10,000 newborn babies are reported as screening positives (18, 19). These are referred to hospital and undergo clinical pediatric evaluation and venous blood sampling for flow cytometric quantification of lymphocyte subsets in order to reach a diagnosis.

Disease causing variants in more than 40 different genes may cause SCID or severe T cell deficiency (20). Knowledge of the specific molecular genetic cause of the immunodeficiency may direct individualized therapy and define the preconditioning regimen for the lifesaving HSCT or thymic transplantation (21). The molecular detection rate for SCID is high, in contrast to other primary immunodeficiencies (22, 23). Atypical, variant and “leaky” SCID are characterized by diminished T cell immunity with low, but non-zero TRECs, with a few exceptions (*ZAP70, ORAI1*, or MHC class II deficiencies) (24). Atypical SCIDs are caused by pathological, but often less deleterious, variants in the same genes as classical SCIDs (4, 24, 25). Therefore, we chose to test the utility of including molecular testing *within* the NBS laboratory's algorithm for SCID. Others have in a retrospective study reported the feasibility of a similar strategy (26), while of ethical reasons we chose a prospective approach. Furthermore, we wanted to establish a pipeline for reliable and rapid SCID screening which could be implemented in the ordinary NBS program.

## Methods

The *prospective* part of the study “NBS for SCID” was designed as a research project and required *written* consent for participation. Parents of newborns born in six selected hospitals in Norway were offered SCID screening of their children. The SCID testing was performed using a single 3.2 mm punch from the same dried blood spot (DBS) filter card (Perkin Elmer 226 specimen collection device or 903 cards from Eastern Business Forms) as the other tests in the ordinary NBS program. When the written consent was received at the national screening unit, identifiers were manually checked and registered. Corresponding DBS samples were selected from the biobank for punching, DNA extraction, and further molecular testing. This pilot study was approved by the Regional Ethical Committee (REC number 2014/128, NBS for SCID) and was conducted in the period Sept 22nd 2015 to Dec 31st 2017. In parallel, we performed a *retrospective* study using samples from patients with primary immunodeficiencies (PIDs) with known and unknown molecular diagnoses to evaluate our methods, establish cut-off levels, and to determine methodological sensitivity and specificity for these disorders (SCID and T cell lymphopenias). It also enabled us to explore the utility and efficacy of 2nd tier NGS integrated in NBS for SCID. The *retrospective* studies were part of the project *Identification of genetic causes of primary immunodeficiency and immunodysregulation using high-throughput sequencing* (REC number 2014/1270) (22). This protocol allowed for testing of individuals with clinically suspected SCID and PID but born at hospitals not included in the pilot. When SCID screening was implemented nationwide in the Norwegian NBS program (January 1st 2018), parental consent was based on *informed* (not written) consent. The parents of the children with SCID and T cell deficiencies identified in the national screening program, in the prospective pilot, and in the retrospective study (Tables 1–**4**), have given written consent to the publication of the medical information included about their child.

**Table 1:** New SCIDs and severe T cell deficiencies identified on newborn screening.

**Patient ID**	**Pilot project**			**National screening**					
	**SCID_1**	**SCID_2**	**SCID_3**	**SCID_4**	**SCID_5**	**SCID_6**	**SCID_7**	**CID_1**	**CID_2**
Symptoms at time of diagnosis	Healthy	Healthy	Skeletal dysplasia, total Hirschprung	Healthy	Healthy	Microcephaly SGA	Healthy	Healthy, Thymic aplasia	Hydrops fetalis, congenital, heart disease, arthrogryposis
Gender	Male	Male	Female	Male	Male	Female	Male	Female	Male
GA w	42	41	37	40	39	40	Male	41	28
BW g	3,592	3,588	2,152	3,618	2,515	2,855	41	4,065	1,330
Mean TRECs/μl	0.48	9.7	0	0	2	1.2	0	11.3	2.12
TREC results avaliable	Day 16	Day 12	Day 11	Day 4	Day 13	Day 6	Day 4	Day 8	Day 4
Age at molecular diagnosis	Day 22	Day 17	After death	Day 8	Day 15	Day 8	Day 6	NA	Prenatal test
Gene	*IL2RG*	*RAG2*	*RMRP*	*DCLRE1C*	*JAK3*	*NBN*	*IL2RG*	none	Trisomy 21
SNV/CNV	c.[359dupA];[0]	c.[1367C>T] HOM	n.[71A>G] HOM	c.[82C>G] HOM	c.[1767C>T]; [2077C>A]	c.[657_661del] HOM	c.[371T>C];[0]	No finding	47, XY, +21
protein	p.Glu121Glyfs*47	p.Ala456Val	NA	p.Ala28Pro	p.(Gly589=); (Pro693Thr)	p.Lys219Asnfs*16	p.Leu124Pro	NA	NA
Refseq	NM_000206.2	NM_000536.3	NR_003051.3	NM_001033855.2	NM_000215.3	NM_002485.4	NM_000206.2	NA	NA
Methods	PIDv2 gene panel	PIDv2 gene panel	PIDv2 gene panel	NBSv2 gene panel	NBSv2 gene panel	NBSv2 gene panel	NBSv2 gene panel	NBSv2 and PIDv2, clinical WES trio and aCGH	Prenatal: Trisomy test and aCGH Postnatal: NBSv2 and clinical WES
Outcome	HSCT, Successful	HSCT, Successful	Deceased 1 month old	HSCT, Successful	HSCT, Successful	Antimicrobial prophylaxis, HSCT considered	HSCT, Successful	Clinical follow-up only, thymus transplantation considered	Deceased 1 month old

*aCGH, array comparative genomic hybridization chromosomal/chromosomal microarray; BW, Birth weight; CNV, Copy number variation; g, gram; GA, Gestational age; NA, Not applicable; NBS, Newborn screening; NBSv2, Newborn screening gene panel version 2; PIDv2, Primary immunodeficiency research panel version 2; PID, Primary immunodeficiency; RefSeq, The National Center for Biotechnology Information Reference Database; SCID, Severe combined immunodeficiency; SNV, Single nucleotide variant; TREC, T-cell receptor excision circles; Trio-test; testing the child and both parents in comparison; WES, whole exome sequencing; w, week*.

In many countries NBS is mandatory, while in Norway NBS is voluntary and based on informed but not written, consent from one of the parents, usually the mother. The parental consent has in practice been obtained similar to the “informed compliance method” described by Kelly et al. (27). Historically, it has been a high (99.9%) participation in the Norwegian NBS program (28). Blood samples from a heel prick of newborn babies are collected on filter cards between 48 and 72 h after birth and sent by overnight express mail. One centralized laboratory, The Norwegian National Newborn Screening Unit performs all analyses. The screening unit is located at the largest University hospital and main pediatric referral center in the country.

## DNA Extraction

DNA was extracted from a 3.2 mm punch of the DBS sample card collected in the ordinary routine NBS program. Samples were punched from the filter card using a Panthera-Puncher 9 (Perkin Elmer, Turku, Finland). The manual method for DNA extraction from filter card blood is modified from Heath et al. (29), and published in detail elsewhere (28). Briefly, the punch was washed with 150 μL DNA Elution solution Qiagen (S2) at 60°C with continuous shaking, followed by elution in 100 μL S2 at 99.5°C for 30 min. One 3.2 mm punch contains on average 3 μL blood, and the extraction method described yields approximately 30 ng DNA from each punch.

## TRECs and β-Actin

**Figure 1:**
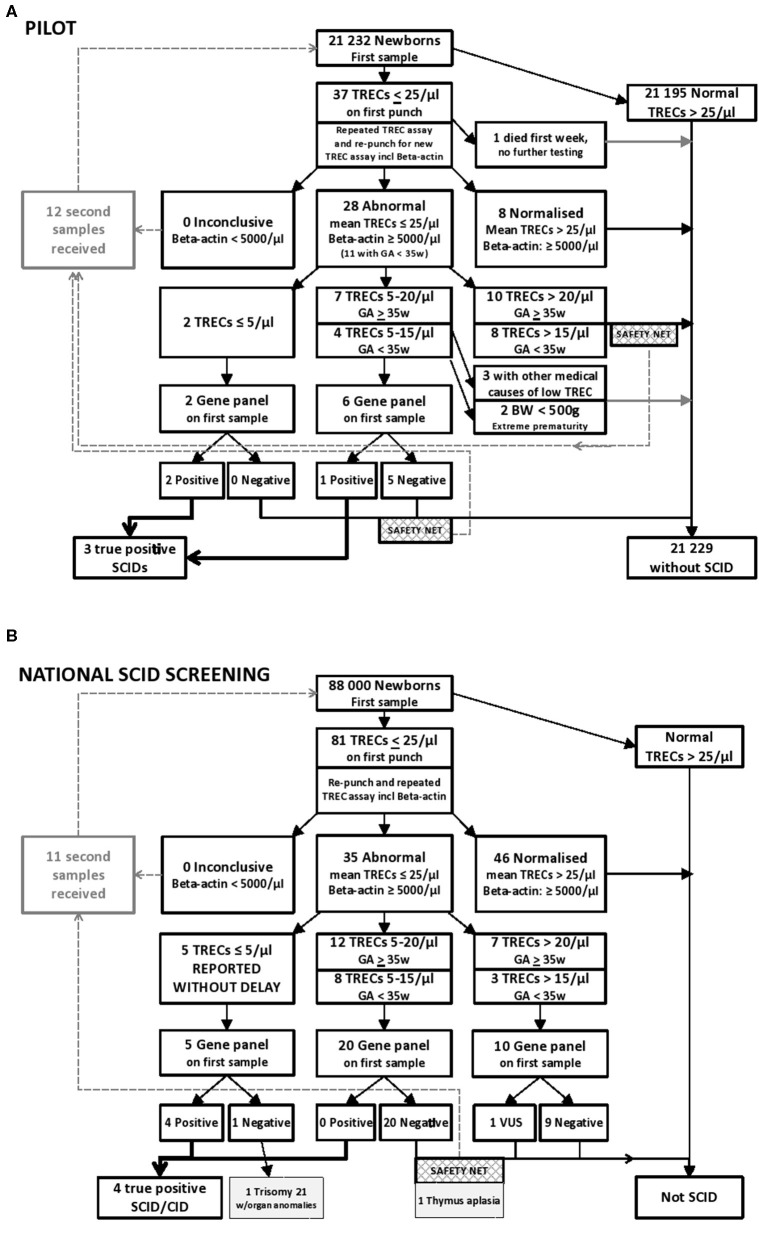
**(A)** Results from the prospective pilot study with NGS gene panel testing integrated in the NBS laboratory algorithm for SCID and other T cell lymphopenias. Samples below 25 TRECs/μL were re-run and re-punched for one new TREC analysis. Samples with normal levels of β-actin (≥ 5,000/μL) and mean TREC levels below 20 TRECs/μL were NGS gene panel tested, dependent on gestational age (GA), birth weight (BW), and information in the baby's medical record. Out of the samples tested in the prospective pilot project, three individuals were identified with SCID. One of them had low intermediate TREC values, consistent with a “leaky” SCID. **(B)** Results from 20 months nationwide screening with rapid NGS gene panel testing integrated in the NBS laboratory algorithm for SCID and other T cell lymphopenias. Samples below 25 TRECs/μL were re-punched and TREC analyses repeated twice on DNA from the new punch. Samples with normal levels of β-actin (≥ 5,000/μL) and mean TREC levels below 25 TRECs/μL were NGS gene panel tested. Out of the samples tested in this nationwide screening between January 2018 to August 2019, 5 had TRECs below 5/μL, and four individuals were identified with severe primary immunodeficiency. The last one had Trisomy 21 with multiple anomalies. As a “safety net” a second DBS sample was requested if NGS was negative when TREC levels were below 20 TRECs/μL (15 TRECs/μL for prematures), which allowed for detection of one individual with congenital thymic aplasia.

**Figure 2:**
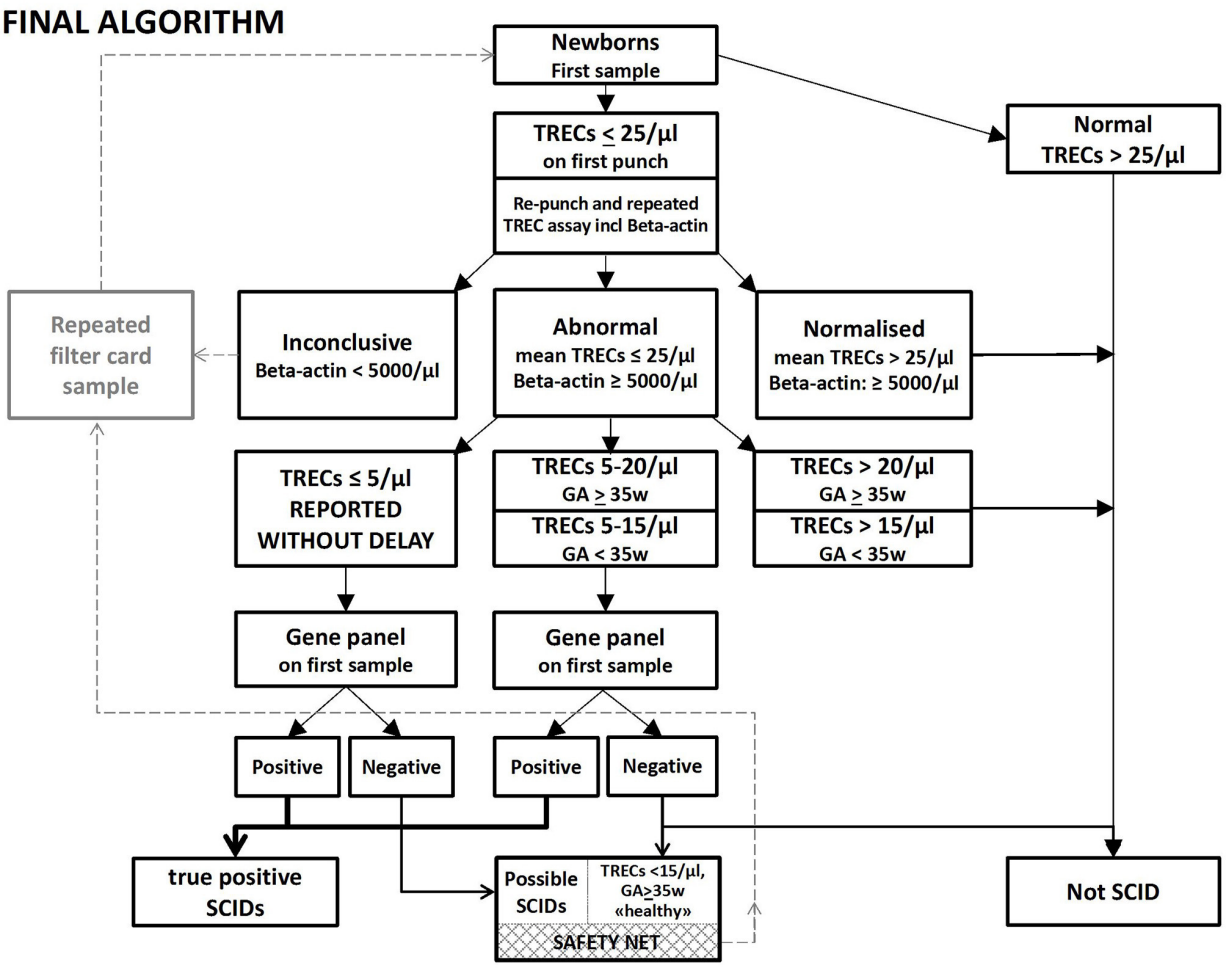
Rapid NGS gene panel testing integrated in the NBS laboratory algorithm for SCID and other T cell lymphopenias, Final algorithm. Samples below 25 TRECs/μL are re-punched and TREC analyses repeated twice on DNA from the new punch. A new DBS sample is requested if low levels of β-actin (<5,000/μL) are found. Samples with TRECs below 20/μL (15/μL for prematures) are immediately NGS gene panel tested. Samples with TRECs below 5/μL are reported without delay, regardless of the NGS findings. If TRECs are below 15/μL and NBS-NGS gene panel negative in an apparently healthy child with normal weight born to term, a second DBS sample is requested as a “safety net.” And if the TREC values are low on the second sample as well, the pediatric immunologist will be alerted, and the child followed further up with clinical investigations.

TRECs and β-actin levels were quantified by qPCR on ViiA7 and QuantStudio 7 (Applied Biosystems/Thermo Fisher Scientific, CA, USA) real-time PCR systems. TREC and β-actin were analyzed in a final volume of 20 μL containing 10 μl PerfeCTa qPCR Toughmix (2x, Quanta Biosciences), 0.8 μl BSA (10 mg/ml) and the following primer sequences: 0.5 μL TREC Forward 5′-CAC ATC CCT TTC AAC CAT GCT-3′ (20 μM), 0.5 μL TREC Reverse 5′-GCC AGC TGC AGG GTT TAG G-3′ (20 μM), 0.2 μL TREC Probe: 5′-FAM-ACA CCT CTG GTT TTT GTA AAG GTG CCC ACT-3′-TAMRA (15 μM), or ACTB primers: 0.5 μL β-actin Forward 5′-ATT TCC CTC TCA GGC ATG GA-3′ (10 μM), 0.5 μL β-actin Reverse 5′-CGT CAC ACT TCA TGA TGG AGT TG-3′ (10 μM), 0.2 μL β-actin Probe: 5′-FAM-GTG GCA TCC ACG AAA CTA-3′-TAMRA (15 μM). To the TREC assay 8 μl DNA was added. To the β-actin assay 4 μL DNA and 4 μl nuclease-free water were added. The reactions were carried out with an initial step at 50°C for 2 min, a denaturation stage at 95°C for 10 min followed by 45 cycles at 95°C for 30 s, and 60°C for 60 s. A calibration curve was created from a TREC plasmid generated and kindly provided by Douek et al. (30). TREC plasmid concentration was determined by Nanodrop (Spectrophotometer ND-1000), and an 8 point standard curve was constructed after 2-fold serial dilutions in dilution solution (Qiagen Generation solution 2 containing tRNA 50 ng/μL). All the qPCR assessments fulfilled the quality requirements of similar slopes and with R^2^ values > 0.975. β-actin was used as a reference gene to assure adequate DNA extraction, and β-actin was only analyzed in samples with TREC values below cut-off values. Our TREC values per μl were based on the assumption that a 3.2 mm punch contains 3 μL of blood. Cut-off value was set to 25 TRECs/μL, and only samples below this initial cut-off were re-run. Samples with normal levels of β-actin (≥ 5,000/μL) and TREC levels in duplicate below 20 TRECs/μL were NGS gene panel tested, dependent on gestational age (GA), birth weight (BW), and information in the baby's medical record (The algorithms are presented in Figures 1A,B, 2).

## NGS Gene Panels

For the specific SCID gene panel test used in the retrospective study, DBS derived DNA (1.5–1.8 ng) was analyzed using the Ion AmpliSeq library kit with the Thermo Fisher predesigned gene panel PIDv1 containing 266 primary immunodeficiency disease genes, and then sequenced on a benchtop ION-PGM (Thermo Fisher Scientific). The PIDv1 gene panel was upgraded at our request by the vendor to PIDv2 with better coverage of certain critical regions such as *ADA* exon 1 (See Discussion). PIDv2 was used as the 2nd tier DNA assessment in the prospective pilot study. The annotated variant calling file (vcf) was filtered in Ion Reporter^TM^ Software to show only variants in the genes relevant for SCID and severe T cell deficiencies. For copy number variant (CNV) detection in the NGS data the Ion Reporter's Confident CNVs-CNVs Only filter was used, and manual visualization of the genes of particular interest. The BAM files were visualized in Integrative Genomics Viewer (IGV) (31) and Alamut Visual (v.2.11, Interactive Bioinformatics, France). Variant evaluation was performed according to the ACMG guidelines (32). The assumed pathogenic gene variants identified by NGS were confirmed using Sanger sequencing, and segregation testing of the parents was performed. In a further development we used the Ion AmpliSeq On-Demand pipeline (Thermo Fisher Scientific) for customized design and synthesis of an NBS-dedicated multiplexed gene panel. This gene panel includes genes for SCID, severe T cell deficiencies and congenital bone marrow failure, genes for the metabolic disorders included in our national NBS program and in addition genes for the US' recommended uniform screening program (RUSP) disorders and their differential diagnoses. This NBS-NGS core panel was designed as a universal newborn screening panel, to be used in the nationwide SCID screening and as 2nd tier DNA testing in general in the NBS program, and the 160-gene list for NBSv1 and upgraded version NBSv2 and NBSv3 containing 186 genes (60 related to SCID/CID/T cell deficiency) are provided as Supplemental Table S7 in the Supplementary Material. Primers have been designed to provide amplicons (average 200 bp) with 99% coverage of coding sequence and a minimum of 10 bp flanking regions of associated introns.

## Follow-Up of Screening Positives

Samples with the lowest TRECs (≤ 5/μL) were regarded as screening positive and immediately reported. For the others with intermediate low TRECs (5–20/μl) only those with molecular confirmation of disease (defined as ACMG class 4 or 5, or a class 3 variant in trans with a 4–5) (32), were regarded as screening positives. After a phone call to parents, the screening positive newborns were admitted to the pediatric immunologists at Oslo University Hospital who coordinated further clinical and laboratory work-ups of the children and serology testing of the mother, according to the agreed protocol: The child was isolated and hygiene advice given to the family awaiting the results of the clinical work-up and new blood samples. Breastfeeding was discontinued (milk production sustained by breast-pumping) until the maternal CMV status was determined as described below. After verification of the SCID with flow cytometry, prophylaxis with antibiotics, antifungals, antivirals, and immunoglobulin infusions was initiated awaiting HSCT.

## Flow Cytometry

Flow cytometric assessments with quantification of absolute counts for T cell (CD3, CD4, CD8), B cell (CD19), and NK cell (CD16/CD56) subsets were performed in EDTA-blood from the child (See Supplemental Tables S1, S5). Absolute counts were analyzed using Multitest 6-color TBNK including Truecount beads according to the manufacturers' instructions (BD Bioscience, San Jose, CA). Further analyses of T cell subpopulations were done to identify recent thymic emigrants (RTE), naïve and memory CD4+ T cells, and if enough sample material was available, additional subpopulations of T and B cells were analyzed. EDTA blood was incubated with optimally titrated antibodies for 15 min at room temperature, followed by erythrocyte lysis using BD FACS Lysing Solution (Beckman Dickinson, San Jose, CA). For B cell analysis, the blood samples were washed twice before incubation with antibodies. Data acquisition was performed on a Gallios Flow Cytometer (Beckman Coulter, San Diego, CA). The following antibodies were used: CD31, CD45RO, CD28, CD45RA, CD127, CD19, CD27; Becton Dickinson, CD4, CD8, CD3, CD25, CD38, IgM, IgD; Beckman Coulter, TCR alfa/beta, CD21; R&D Systems (Minneapolis, MN), CXCR5; eBioscience (San Diego, CA), CD45; Invitrogen (Waltham, MA), CD27; Dako (Glostrup, Denmark). T cells were gated as CD3+ and further as naive CD4+ (CD4+, CD45RA+), recent thymic emigrants (CD4+, CD45RA+, CD31+), CD4+ memory (CD4+, CD45RO+), follicular like CD4+ (CD4+, CD45RO+, CCR5+), regulatory T cells (CD4+, CD25++, CD127–), naive CD8+ (CD8+, CD27+, CD28+), CD8+ early effector memory (CD8+, CD27+, CD28–), CD8+ late effector memory (CD8+, CD27–, CD28–). B cells were gated as CD19+ and further subclassified as naive (IgD+, IgM+, CD27–), IgM memory (CD27+, IgD+, IgM+), class switched (CD27+, IgM–, IgD–), plasmablasts (CD19+dim, CD27++, CD38++), transitional (IgM++, CD38++, CD24+), and CD21 low B cells (CD38 low, CD21 low).

## CMV Analyses

CMV serostatus of the mother was investigated immediately after positive SCID screening not awaiting the molecular confirmation when TREC was ≤ 5/μL. The Abbott Architect chemiluminescent microparticle immunoassay (CMIA) (Abbott Laboratories, USA) was used for the qualitative detection of IgM and IgG antibodies to CMV. If maternal serology was CMV positive, defined as IgM and/or IgG positive, the CMV viral load in the child's urine and blood were measured after DNA extraction with MagNa Pure LC 96 (Roche Diagnostics, USA). An in-house method using a quantitative real-time PCR (LightCycler® 480, Roche Diagnostics, USA) with the conserved area of the polymerase gene UL54 as the target was used to quantify the CMV viral load. The detection limit was set to 36 IU/mL in each sample, and the quantification range to between 200 and 10,000,000 IU/mL. For comparison, the CMV viral load at birth was measured with the same qPCR method using DNA extracted from two new punches from the child's initial NBS filter card. This extraction method is described elsewhere (33).

## Results

We have explored the utility of rapid NGS integrated in the NBS laboratory algorithm for SCID. Out of the 21,232 samples tested in the prospective pilot project, three individuals were identified with SCID: on day 16, 13, and 11 after birth, respectively; one *IL2RG*, one *RAG2*, and one *RMPR* related SCID (Table 1 and Figure 1A). Filter cards were received in the lab on day 4, 6, and 3 after birth, but the consent forms were sent separately, and since this was initially a research project and not part of the ordinary newborn screening, TREC testing was not performed until day 15, 12, and 11. The child with *RMRP*-SCID had complete Hirschsprung disease and died at 1 month of age. For the two others, within 4–5 days after TREC-testing and DNA extraction from the original NBS DBS sample, the sequencing results were available, utilizing NGS with Ion AmpliSeq^TM^ PID panel, and the molecular diagnosis was received at day 22 and day 17, respectively (Table 1). The *RAG2-*SCID patient had low intermediate TREC values, mean 9.7/μl, consistent with a leaky SCID. The molecular findings directed follow-up and therapy: the *IL2RG*-SCID underwent early hematopoietic stem cell transplantations (HSCT), without any complications; the leaky *RAG2-*SCID received prophylactic antibiotics, antiviral and antifungal treatment, immunoglobulin infusions, and was transplanted later, at 1 year of age (Supplemental Table S1). Both mothers were instructed (day 16 and day 17) to avoid breastfeeding their child for a short period of time before the maternal CMV status was determined by serological testing. One of the mothers was CMV positive (IgG positive, IgM negative, day 20) and breastfeeding was discontinued. The other mother was CMV negative (day 25) and breastfeeding was immediately re-started. Neither of these two babies became CMV infected prior to transplantation. Familial segregation testing confirmed the expected inheritance. The maternal uncle of the *IL2RG*-SCID patient had died of an infection in early childhood, and the maternal grandmother was a carrier of the *IL2RG* variant. Further genetic carrier testing has been offered for the potential carriers in the family. The parents of individual SCID_2 were confirmed carriers of the *RAG2* variant, and the SCID_3 individual's parents were both confirmed carriers of the *RMRP* variant (Table 1), hence, the apparent homozygous variants identified were located in trans alleles in these children, excluding allelic drop-out.

Only 37 (0.17%) of the 21,232 individuals in the pilot study had TRECs below 25/μl on the initial test (Figure 1A). Retesting was performed using both the original DNA-extract and DNA obtained from re-punching of the dried blood spot. Eight samples with TREC 0 on the first run had TREC-levels above 100/μl on the re-runs (data not shown) and were regarded as normal/negative. The 24 individuals in the pilot study with the lowest TREC values after retesting are presented in detail in Supplemental Table S2. Among these were nine premature babies with low birth weight, eight with intestinal malformations, and six with congenital heart disease either as a single feature or in combination with other comorbidities or prematurity. Altogether 21 of the 24 newborns with the lowest TRECs were hospitalized in neonatal intensive care units (NICU) (Supplemental Table S2), 20 of them at Oslo University Hospital (OUS). The PID panel was only run on selected patient samples. Genetic testing was not performed when there was another clear medical explanation for the low TREC such as intestinal malformation, multiple transfusions, extreme prematurity or intensive care after surgery (34). Out of the 21,232 samples TREC tested in the prospective pilot, nine underwent genetic testing as part of the screening (0.04%) (Figure 1A). Re-draws were requested in only 12 individuals (Supplemental Table S2). After the pilot, the cost of the total SCID screening reagents, including NGS of the selected cases with the lowest TREC values, was estimated to be 10 USD per sample.

The overall mean value in the pilot study was 285 TRECs/μl (5^th^-95th centile range: 92.6–614 TRECs/μl), and median 244 TRECs/μl, when samples with values below 25/μl on the initial test were excluded. A significant correlation between TRECs and gestational age and birth weight was observed (Supplemental Figure S1). Premature babies and those with low birth weight had lower TREC values. The male newborns had slightly higher mean birth weight and lower TRECs values as compared to the girls (Supplemental Figure S2).

The Norwegian newborn screening unit is located at the hospital performing all HSCTs in children in Norway, and OUS is also the main referral hospital for critically ill neonates and infants. Six children, born during the period of the pilot study at hospitals not included in SCID screening, were referred to our clinic at 1–6 months of age based on symptoms such as infections and poor growth or dysmorphology with suspected immunodeficiency (Table 2). Their original newborn dried blood spots as well as a new sample were tested, and all had low TREC-levels and SCID-features at the time of referral. All except one had TRECs below the initial cut-off value (25/μl) at birth (Supplemental Figure S2B). A single patient (PID_6, Table 2) had TRECs of 72/μl at birth, but low TRECs (12/μl) when tested at 6 months. Gene panel testing on DBS DNA identified a *IKZF1*-related lymphoproliferative variant c.[476A>G];[=], p.Asn159Ser (35, 36). This variant occurred *de novo*. Directed by the genetic finding (35, 36), as the specific variant was reported to cause lymphoproliferative disorder, this child underwent HSCT, and is currently healthy 1 year after transplantation (37), demonstrating the utility of combining TREC testing and NGS as a diagnostic tool beyond newborn screening. However, in two of the other children (PID_1 and PID_2, Supplemental Table S3), both born small for gestational age (and named “SGA and leukopenia” in Supplemental Figure S1B), we were not able to identify the molecular cause of their disease. The last three had CHARGE syndrome, Down syndrome, and DiGeorge syndrome, respectively (PID_3, PID_4 and PID_5 in Table 2 and Supplemental Table S3).

**Table 2:** New severe PIDs, debut 3–6 months, not picked up on newborn screening since born outside pilot test region.

**Patient ID**	**TRECs and PIDv2 panel testing on the original DBS and the new sample**					
	**PID_1**	**PID_2**	**PID_3**	**PID_4**	**PID_5**	**PID_6**
Year	2016	2016	2017	2017	2017	2017
Symptoms at time of diagnosis	SGA, microcephaly, transient lymphopenia, persistent neutropenia and low number of platelets	SGA, microcephaly, failure to thrive, pigment patches skin	Heart defect, choanal atresia, coloboma, infections	Heart defect, prematurity, Downs syndrome	Heart defect, dysmorphic features	Infections, failure to thrive
Gender	Female	Male	Male	Male	Male	Female
GA w	35	36	36	33	34	40
BW g	1,920	1,534	2,445		1,570	3,430
TRECs/μl at birth	4.69	1.9	0	9.7	15.7	71.8
Repeated TRECs/ul (age)	23.8 (3 months) 0 (12 months)	0 (4 months)	0 (1.5 months)	0 (2 months)	NA	12.2 (6.8 months)
Gene	unknown	unknown	*CHD7*	Trisomy 21	*TBX1*	*IKZF1*
SNV/CNV	NA	NA	c.[5833C>T];[=]	47, XY, +21	22q11.21 del	c.[476A < G];[=]
Protein	NA	NA	p.Arg1945*	NA	NA	p.Asn159Ser
Refseq	NA	NA	NM_017780.3	NA	NA	NM_006060.4
Methods	PIDv2 gene panel, WES/WGS trio	PIDv2 gene panel, WES/WGS trio	Sanger	Trisomy test	MLPA, Trisomy test	PIDv2 gene panel
Treatment and outcome	Deceased at 1 ½ years of age	HSCT, Successful	Prophylactic antibiotics, antifungal, antiviral and ScIg therapy	Heart surgery. Lymphopenia 1.9 × 10^9^/L at 1 years of age, but no recurrent infections	Prophylactic antibiotics	HSCT, Successful

*aCGH, array comparative genomic hybridization chromosomal/chromosomal microarray; BW, Birth weight; CNV, Copy number variation; g, gram; GA, Gestational age; HGNC, The HUGO Gene Nomenclature Committee; HSCT, Hematopoietic Stem Cell Transplantation; MLPA, multiplex ligation-dependent probe amplification; NA, Not applicable; NBS, Newborn screening; ND, Not detected; PIDv2, Primary immunodeficiency research panel version 2; PIDs, Primary immunodeficiencies; RefSeq, The National Center for Biotechnology Information Reference Database; SCID, Severe combined immunodeficiency; ScIg, Subcutaneous Immunoglobulin; SGA, Small for gestational age; SNV, Single nucleotide variant; TREC, T-cell receptor excision circles; Trio-test; testing the child and both parents in comparison; WES, whole exome sequencing; WGS, whole genome sequencing w, week*.

*Resources: Gene names according to HGNC, https://www.genenames.org/*.

*Gene variant nomenclature according to the HGVS recommendations, http://www.HGVS.org/varnomen*.

During the pilot study time period ~130,000 children were born in Norway (https://www.ssb.no/fodte/; 56,633 year 2017, 58,890 year 2016, and 13,210 Oct-Dec 2015). The incidence of SCID (based on the *IL2RG-, RAG2-, RMPR-*, and the *CHD7*-SCID, plus the child with the bone marrow failure and yet unidentified DNA-repair disorder, PID_2 in Table 2) was 1:26,000.

With regard to the 2nd tier NGS results in the *retrospective* study we were able to confirm the following molecular findings: *IL2RG* hemizygous c.924+5G>A (NM_000206.2), *JAK3* compound heterozygous c.1837C>T; c.1695C>A (NM_000215.3)*, IL7R* homozygous c.707-2A>G (NM_002185.3)*, LIG4* compound heterozygous c.1341G>T; c.482delC (NM_002312.3), *PGM3* homozygous c.737A>G (NM_015599.2), and the *RECQL4* heterozygous variant c.2269C>T (NM_004260.3), but not the intronic c.3056-3C>A which had been identified by WES and published earlier (22), since the predesigned gene panels had no amplicon which included this particular region of *RECQL4*. We were not initially able to detect any *ADA* sequence alteration(s) in the ADA*-*SCID sample. The patient had parents of Somali ethnicity, and by studying the raw BAM file it turned out that the PIDv1 gene panel was missing exon 1 where the Somali ADA founder mutation is located. Hence, PIDv2 was developed and used as a 2nd tier DNA assessment in the pilot project and the retrospective study (Table 3 and Supplemental Table S4). DiGeorge syndrome deletion 22q11 was tested by *TBX1* dosage using bioinformatic interpretation of NGS data. Of note, 55% (five out of nine) of the known Ataxia-Telangiectasia patients would have been detected on initial screening using a TREC cut-off below 25 TRECs/μl (Table 4). As a result of our pilot project and retrospective studies, the Norwegian government and health authorities decided to mandate nationwide NBS for SCID.

**Table 3:** Retrospective TRECs and NGS testing in known PIDs using DNA from the original newborn screening DBS.

**Sample ID**	**Individuals with known SCID or severe T-cell deficiency**									**Other PIDs**	
	**KID_1**	**KID_2**	**KID_3**	**KID_4**	**KID_5**	**KID_6**	**KID_7**	**KID_8**	**KID_9**	**KID_10**	**KID_11**
Year	2012	2010	2010	2015	2012	2014	2015	2006	2009	2009	2012
Gender	Male	Female	Female	Female	Female	Male	Male	Female	Male	Male	Female
GA w	37	34	38,5	42	40	42	39	38	39	40	38
BW g	2,954	1,999	2,618	3,360	4,135	4,420	2,484	NA	3,445	3,775	2,950
TRECs/μl	0	0	0	0	11	0	0	0	0	25.5	60
Gene	*IL2RG*	*LIG4*	*IL7R*	*ADA*	*PGM3*	*JAK3*	*TBX1*	*TBX1*	*TBX1*	*IKZF1*	*RECQL4*
SNV/CNV	c.[924+5G>A]; [0]	c.[1341G>T]; [482delC]	c.[707-2A>G] HOM	c.[7C>T] HOM	c.[737A>G] HOM	c.[1837C>T]; [1695C>A]	22q11.21 del	22q11.21 del	22q11.21 del	c.1618388_589 +2308del (16.8 kb del exons 4-5)	c.[2269C>T]; ND (22)
Protein	Splice defect	p.Trp447Cys; p.Ala161Valfs*6	Splice defect	p.Gln3*	p.Ala246Gly	p.Arg613*; p.Cys565*	loss	loss	loss	Inframe deletion	p.Gln757*
RefSeq	NM_ 000206.2	NM_ 002312.3	NM_ 002185.3	NM_ 000022.2	NM_ 015599.2	NM_ 000215.3	NM_ 080647.1	NM_ 080647.1	NM_ 080647.1	NM_ 006060.5	NM_ 004260.3
Panel	PIDv1	PIDv1	PIDv1	PIDv2	PIDv2	PIDv1	PIDv2	PIDv2	PIDv2	PIDv2	PIDv1 and PIDv2

*BW, Birth weight; CNV, Copy number variation; DBS, dried blood spot; del, deletion; g, gram; GA, Gestational age; HOM, Homozygous; HGNC, The HUGO Gene Nomenclature Committee; NBS, Newborn screening; ND, Not detected; NGS, Next generation sequencing; PIDs, Primary immunodeficiencies; PIDv2, Primary immunodeficiency research panel version 2; RefSeq, The National Center for Biotechnology Information Reference Database; SCID, Severe combined immunodeficiency; SNV, Single nucleotide variant; TREC, T-cell receptor excision circles; w, weeks*.

*Resources: Gene names according to HGNC, https://www.genenames.org/; Gene variant nomenclature according to the HGVS recommendations, http://www.HGVS.org/varnomen*.

*Extended information available in Supplemental Table S4*.

**Table 4:** Retrospective TRECs and NGS testing in known PIDs using DNA from the original newborn screening DBS.

**Individuals with known ataxia telangiectasia**									
**Sample ID**	**AT_1.1**	**AT_1.2**	**AT_2**	**AT_3**	**AT_4**	**AT_5**	**AT_6**	**AT_7**	**AT_8**
Year	2008	2011	2010	2010	2011	2011	2013	2014	2016
Gender	Male	Male	Female	Female	Male	Male	Male	Female	Female
GA w	36	39	40	40	41	41	39	40	40
BW g	3,015	3,650	3,196	3,570	2,815	4,180	3,020	3,490	2,959
TRECs/μl	3.9	2.8	15.7	7	21.7	92.8	67.2	202.7	27.2
Gene, RefSeq	*ATM*, NM_000051.3								
SNV/CNV	c.[6047A>G] HOM	c.[6047A>G] HOM	c.[3245_3247delATC insTGAT] HOM	c.[3245_3247delATC insTGAT];[6679C>T]	c.[3245_3247delATC insTGAT] HOM	c.[1564_1565delGA]; [9023G>A]	c.[3245_3247delATC insTGAT];[5712dupA]	c.[3245_3247delATC insTGAT];[8030A>G]	c.[5932G>T]; [9126delC]
protein	p.Asp2016Gly	p.Asp2016Gly	p.(His1082Leufs*14)	p.(His1082Leufs*14); p.Arg2227Cys	p.(His1082Leufs*14)	p.Glu522Ilefs*43; p.Arg3008His	p.(His1082Leufs*14); p.Ser1905Ilefs*25	p.(His1082Leufs*14); p.Tyr2677Cys	p.Glu1978*; p.Asn3044Ilefs*31
Panel	PIDv2	PIDv2	PIDv2	PIDv2	PIDv2	PIDv2	NBSv2	NBSv1	PIDv2

*BW, Birth weight; CNV, Copy number variation; DBS, dried blood spot; del, deletion; del, deletion; dup, duplication; g, gram; GA, Gestational age; HOM, Homozygous; HGNC, The HUGO Gene Nomenclature Committee; ins, insertion; NBSv1, Newborn screening gene panel version 1; NGS, Next generation sequencing; PIDv2, Primary immunodeficiency research panel version 2; RefSeq, The National Center for Biotechnology Information Reference Database; SNV, Single nucleotide variant; TREC, T-cell receptor excision circles; w, week*.

*Resource: Gene variant nomenclature according to the HGVS recommendations, http://www.HGVS.org/varnomen*.

## Proof-of-principle for Rapid NGS in NBS

When NBS for SCID was implemented nationwide in the ordinary screening panel, the customized targeted NBS-NGS core panel (See Methods and Supplemental Table S7) was used. The test algorithm and results of the first period of routine SCID screening are presented in Figure 1B. The first SCID patient was identified within 4 days after birth with zero TRECs/μl on 3 separate punches (Blood sample collected day 2, arrived at the central lab and the 1st TREC test performed day 3, two new punches and re-run TREC tests performed day 4, and β-actin tested in parallel). When the local hospital and the parents were informed about the SCID-suspicion, at day 4; the mother was immediately asked to discontinue breast-feeding. The mother was serological CMV tested, and at day 9 she turned out to be CMV positive (IgG pos/IgM neg, Supplemental Table S5), hence, breast-feeding was permanently avoided for this child. This story of the first SCID identified during routine screening (individual SCID_4, Table 1) demonstrates the applicability of rapid NGS integrated in the SCID screening lab testing algorithm: Using DNA from the original newborn screening dried blood spot sample, at day 8, it was clear that the male infant was homozygous for a *DCLRE1C* variant and had Artemis-SCID. The variant NM_001033855.2(DCLRE1C): c.[82C>G](;)[82C>G], p.Ala28Pro had previously been reported in T-B-NK+ SCID (38). A donor search was then initiated. While waiting for the transplantation, the infant was treated with prophylactic doses of antivirals, antifungals and antibiotics, plus immunoglobulin infusions. X rays and CT scans were avoided due to the molecular confirmation of radiosensitive SCID, and he followed a modified preconditioning HSCT protocol (Table 1 and Supplemental Table S5).

Yet another baby born in 2018 (individual SCID_5, Table 1 and Supplemental Table S5) was identified with SCID at day 13 with zero TRECs, and the *JAK3* variants detected by NBS-NGS core panel on day 15. His mother stopped breastfeeding on day 14 and was verified seropositive at day 17, but the baby had by then already acquired a CMV infection: CMV PCR was negative on the initial NBS filter card, while CMV in plasma was >1,000 IU/mL day 14. He then received antiviral treatment in line with recommendations by Vicetti Miguel et al. (3).

Furthermore, reactivation of CMV was observed 2 months post-HSCT with virus load increasing to >100,000 IU/mL. The reason for the increased turn-around-time and late screening in the JAK3 patient were delays both in the mail and the laboratory (Sample had been collected on day 2, but arrived in the NBS lab at day 8, and the TREC results were not ready until day 13, while the *JAK3* variants were identified day 15).

In the last SCID patient identified and reported here (individual SCID_7, Table 1), zero TRECs were detected in the filter card on day 4 after birth, molecular confirmation of *IL2RG* disease was ready on day 6, again demonstrating the value of rapid NGS in NBS. Breast-feeding was stopped immediately after the low TRECs were reported, the mother was tested and found CMV seropositive day 5, and the child was not CMV infected (SCID_7, Supplemental Table S5).

Out of the ~88,000 newborns SCID screened during 2018 and 2019 (from Jan 1st 2018 to Aug 1st 2019) 81 had TRECs below 25/μl on the first test, 35 below 25/μl on the repeated test, and all of these 35 (0.4% of samples) were NGS tested (Figure 1B). Five had TRECs between zero and 5/μl and were immediately reported, and among them, one Nijmegen breakage syndrome (*NBN*) with SCID-like phenotype and three classical SCIDs (Artemis, *JAK3*, and *IL2RG*) were identified (Table 1). The last one with TRECs below 5/μl had Trisomy 21 was born prematurely with multiple anomalies (hydrops fetalis, congenital heart disease and arthrogryposis affecting all extremities), and later died at 1 month of age (CID_2 in Table 1, and in Figure 1B). Regarding the others (*n* = 30), none had definite diagnostic findings on the NBS gene panel testing. Twenty-one of the 30 infants were in NICU at the time of first sampling, and a repeat filter card blood test (redraw) was requested from 12 (received from 11) individuals; of which six had received total parenteral nutrition (TPN), and new samples were requested mainly due to the expectation of TPN interference in the biochemical screening markers for metabolic disorders on the first sample. Despite the lack of gene findings on our NBSv2 panel, redraws were specifically requested from five apparently healthy children born at term with low mean TREC values of 11.2, 11.4, 13.3, 17, and 23.4/μl, respectively (Supplemental Table S6). Four of them had normalized TREC values on the second sample, but one (individual CID_1 Table 1) had consistently low TRECs, no pathogenic gene findings on the NBSv2 or the PIDv2 panel, few T cells on flow cytometry and thymic aplasia on ultrasound. Further genetic testing with WES, chromosomal microarray and MLPA (multiplex ligation-dependent probe amplification) targeting single exons in *CHD7* did not reveal any genetic cause of the missing thymus and T cells (CID_1 in Table 1 and Supplemental Table S5). One child with low TRECs and generalized skin disease, scaly erythroderma, and therefore suspected Omenn syndrome had a homozygous variant of unknown significance (VUS) in *TTCA7*. The variant was evaluated to be a rare variant unrelated to disease, and whole exome sequencing (WES) later identified a homozygous deleterious variant in *SPINK5* causing Netherton syndrome. In another baby with mean TRECs of 9/μl and no findings on the NBS gene panel, WES later identified *PMM2*-CGD. None of these two babies were reported as SCID screening positives (Supplemental Table S6). No other children born within this time period (Jan 2018-Aug 2019) have yet been referred for SCID or severe T cell deficiency besides the ones identified through the national screening program. For the 3 SCID-patients and the Nijmegen breakage syndrome case with SCID-like phenotype identified by screening in the national program the TRECs results were ready at day 4, 13, 4, and 6, respectively, and their molecular diagnosis (*DCLRE1C, JAK3, IL2RG, NBN)* were molecularly confirmed on day 8, 15, 6, and 8 after birth, respectively, using the initial blood spot. Four SCIDs out of 88,000 tested reflects an incidence of 1 per 22,000 live births, 1: 29,300 without the NBN case.

## Discussion

To our knowledge we were the first country in Europe to provide nationwide SCID screening, although 10 years behind the first states in the US (39). Our nationwide SCID screening started 1 year after the Spanish region of Catalonia offered SCID screening to all newborns in their region (8). Catalonia has 7.5 million inhabitants, while Norway has 5.4 million inhabitants. The incidence of SCID in Catalonia was 1:130,000 births after 2 years (8), while we ascertained five times more cases: The incidence of SCID and severe T cell deficiencies was 1:26,000 in the pilot study and 1:22,000 after the screening was made nationwide, 1: 29,300 without the NBN case. A high incidence of SCID and severe T cell deficiencies has been reported in certain populations and ethnic groups (26, 40–42), as well as an increased incidence in the general population after the introduction of screening, contrasting with the pre-SCID-screening estimated incidence from historical data and medical records (43). With the introduction of NBS for SCID in the US, and based on more than 3 million screened babies, the overall incidence was revised to 1:58,000, which was nearly twice their pre-screening estimated numbers (44). In the US there are also population pockets with higher incidence of SCID due to founder mutations (42). The parents of the reported patients in our screening studies were a mixture of ethnic Norwegians and Norwegians with immigrant parents originating from Estonia, Lithuania, Poland, Turkey, and Somalia, which presently reflects the population in our country. Whether immigration has led to a changed incidence of SCID and T cell disorders in Norway remains to be documented, but certain disorders and founder variants such as in ADA-SCID (KID_4, Table 3) may have increased compared to our historical numbers (43). However, precautions need to be taken since our total numbers are small. Our numbers for other severe T cell disorders and atypical SCID are high, but should not be extrapolated to the whole population because of the small screening total and the short observation time. Several years of national screening is needed to provide a more robust estimate of the incidence of SCID and severe T cell disorders in our population. The true incidence of SCID in Norway is nevertheless probably much higher than the numbers estimated from the epidemiological study in Norway (1:100,000 live births) (43). It is unlikely that this is solely explained by the increase in immigrant child births. There is a high likelihood that several pre-screening SCID patients died of infections without receiving a SCID diagnosis (43).

Our national SCID screening program includes rapid and broad integrated molecular diagnostic testing on dried blood spots, and was the first to implement this routine worldwide (26). We have shown that the combination of TREC measurements and high throughput NGS analyses using targeted gene panels in newborn screening is an effective genotype-based approach to molecular diagnosis for affected infants. This combination also increases the diagnostic precision, limits false positives, and minimizes unnecessary contact with the families of healthy newborns; fulfilling a fundamental goal for newborn screening laboratories (45). Early identification of the molecular cause and disease mechanisms enable early protective interventions and targeted or curative therapy. In patients identified with radiosensitive SCID, precautions and modification regarding radiographic imaging and the use of DNA-damaging radiomimetic drugs in pre-treatment conditioning can immediately be addressed, such as in the Artemis-SCID (individual SCID_4, Table 1 and Supplemental Table S5).

CMV can be a serious and fatal infection even in early identified, newborn-screened SCID infants (3, 4). Others have documented that more than 40% develop infections before HSCT (4) such as varicella (23), rubella, EBV, with CMV being the most common. Although patients diagnosed via NBS are less likely to have an infection before HSCT, avoiding post-natal CMV infection remains an important challenge (4, 46) The NBS algorithms that require a second dry blood spot have their final TREC results available only after 3–4 weeks of age (44), limiting the oppurtunities for early CMV intervention. A faster turn-around-time to a definite SCID diagnosis may be the most practical way to reduce CMV exposure time (45), and general viral infection rates (23). Six children had CMV seropositive mothers (one during the pilot and five from routine screening). Four of these individuals were identified early, within the first week of life, and did not acquire a CMV infection. Two infants were identified with SCID as late as 2 weeks after birth, and one of them developed a clinical CMV infection, the child with *JAK3*-SCID and complete absence of T cells. The other child had a leaky *RAG2*-SCID, and a virus protective effect of the residual T cells cannot be excluded. The CMV infection in the *JAK3*-SCID was presumably acquired postnatally, since CMV was not detected in the initial NBS filter card, but was found in plasma at 2 weeks of age. Not all congenital CMV infections are detected by CMV DNA analysis in DBS (47), but the increasing virus load observed in plasma over 2–3 weeks (individual SCID_5, Table 1 and Supplemental Table S5), is in accordance with the known incubation time for CMV infection in infants (48). And maternal transmission through breastfeeding is a plausible explanation. CMV is reactivated in nearly all latently infected mothers and is excreted in the breast milk (3, 48, 49). CMV can also be transmitted via saliva. If the SCID identification through screening proceeds seamlessly and without delay from sample collection to positive screening, and breastfeeding in seropositive mothers is prevented at an early stage, perhaps the most optimal pre-HSCT health condition can be achieved. However, this remains to be proven beyond the four individuals ascertained and reported here, since others have recently reported surprisingly few CMV infected among breast fed in their historical SCID data (50).

A critical step in the implementation of full scale screening was choosing the TREC cut-off and deciding how to handle samples which were positive after 1st tier analysis. With NGS integrated into the NBS algorithm, we chose a 1st tier TREC cut-off value of 25/μl. Other SCID screening algorithms, without NGS, regularly use lower cut-offs, request new samples and/or report more false positives (See paragraph below). Our relatively high TREC cut-off value allowed for identification of leaky SCID such as the *RAG2-*SCID in individual SCID_2 (Table 1 and Supplemental Table S1). The majority of samples with moderately low TREC values (5–20/μL) showed no genetic variants associated with SCID or T cell deficiencies and were therefore not reported. Inevitably, a negative screening result cannot absolutely exclude all combined immunodeficiencies, exemplified by the patient with the *IKZF1* missense variant (PID_6, Table 2) who presented after the newborn period. Not all molecular aberrations are detected or known, shown by the two children born outside pilot region with leukopenia and small for gestational age (PID_1 and PID_2, Table 2), and the apparently healthy child with thymus aplasia born at term with normal birth weights (CID_1, Table 1). Novel genes related to disorders with congenital T cell deficiency are constantly being reported and consecutively included on the updated International Union of Immunological Societies, IUIS' lists of recognized human inborn errrors of immunity (20, 51). Thus, NGS cannot replace TRECs or other NBS markers, and a “safety net” is still needed for those with the lowest TRECs.

The heterogeneity of SCID requires a broad testing approach with large gene panels and ideally the ability to identify structural variants, such as large and small copy number variants (CNVs) (52). Among the few SCID and T cell deficient children we have NBS-NGS gene panel tested here, we have found various immunodeficiencies with multiple genetic causes (*IL2RG, RAG2, RMRP, IKZF1, CHD7, DCLRE1C, JAK3*), with different inheritance patterns (AR, AD, de novo, XL) and mutation types (i.e., CNVs in DiGeorge syndrome, intragenic *IKZF1* deletion and larger chromosomal aberrations such as Trisomy 21). The heterogeneity and the expanding number of immunodeficiency genes therefore require a broad list of genes and regular updates of the panel.

Based on the experiences from the prospective pilot study, the retrospective study and the first period of nationwide SCID screening, we propose the following refined and final algorithm (Figure 2): All samples below initial cut-off 25 TRECs/μL will be re-punched in duplicate. Samples with two new punches with mean TREC levels below 20 TRECs/μL (below 15/μL for premature babies, GA <35 weeks) and normal levels of β-actin proceed to NGS gene panel testing. However, if two out of the three TREC measurements/analyses are below 5/μL, the pediatric immunologist and family should be called immediately, without awaiting the NGS results, breastfeeding stopped, maternal CMV serology checked and flow cytometry performed in the child. Samples with TREC values between 5 and 20/μL—predominantly from premature or NICU infants—where NGS does not reveal any disease-causing mutations, are signed out as normal/negative SCID screens. A “safety net” allows for the collection of a new dried blood spot sample for apparently healthy babies born at term with TRECs 5–15/μL, without pathogenic NGS findings, and with no explanation for the low TRECs such as transient disease, congenital heart defect or intestinal abnormality (Figure 2) (34, 53). These children are followed-up by the pediatric immunology team and further diagnostic and functional testing is performed. With this algorithm, classical SCID will be identified, the time from birth to definitive diagnosis substantially reduced, and the number of recalls and second samples minimized despite our high initial cut-off value. By strictly following this algorithm for the 88,000 screened newborn babies nationwide, all SCID and severe T cell deficiencies would have been identified and only three other redraws requested.

Other programs have reported 0.08–0.4% redraws and recalls (5, 8, 44, 54, 55), and 0.016–0.14% for lymphocyte flow cytometry and consultation (18, 44, 56), while our overall redraw rate was 0.07% (12 second filter card + 3 SCIDs) out of 21 000 in the pilot study and 0.02% (13 second filter card sample requested + 4 SCIDs) out of the 88,000 in the nationwide screening, We could have achieved recalls as low as 0.01% with only 3 “false positives” if we had strictly followed our final algorithm (Figure 2). Many of our second filter card requests were due to the interference of total parenteral nutrition (TPN) with the biochemical screening markers used in NBS for inborn errors of metabolism, not immunodeficiency (Supplemental Table S6).

With this approach we are also able to identify Ataxia Telangiectasia (AT), which was previously reported by Mallot et al. (57) and others (58). We have not identified any AT patient in the pilot, or so far in the nationwide NBS. Norway has a higher incidence of AT as compared to neighboring countries in Europe, and the Norwegian AT founder mutation is expected to have an impact on the number of AT patients identified by NBS in the long run (59, 60). A plan for the diagnostic process, follow-up and care of the family needs to be in place for the AT-group, where there is currently no curative treatment available for the neurological phenotype, and where the clinical signs of neurodegeneration with ataxia usually start at 1 year of age (61). HSCT has been controversial and avoided in this group of DNA repair disorders for many years, but may with modified preconditioning be safely used to cure the immunodeficiency (62). The child identified with Nijmegen breakage syndrome had zero TRECs and a SCID-like phenotype, and HSCT was considered, since it has been performed in other NBN cases (63), but not planned in this case. Others have also identified children with Nijmegen breakage syndrome by SCID screening (64).

The successful integration of NGS within NBS paves the way toward expanding the NBS program to further include other severe and treatable genetic disorders and immunodeficiencies such as severe congenital neutropenia, X-linked and autosomal recessive chronic granulomatous disease and congenital agammaglobulinemia. New screening methods for other PIDs have been developed, with or without DNA-based markers (11, 65–67), but some disorders require a genetic method as the 1st tier test. NBS in Norway is based on informed consent, and allows for *genetic* mass screening of selected disorders to be performed without formal genetic counseling. It has been very useful in cystic fibrosis screening (28), in screening for metabolic disorders with limited numbers of disease genes (68–71), and for SCID screening with multiple disease genes. Analysis of biobanked samples from patients with known PIDs to evaluate the test methods, were performed retrospectively. The pilot was performed prospectively for ethical reasons; we did not want to test for SCID retrospectively with the lost opportunity to intervene and treat the affected newborns. However, the administrative challenge of obtaining *written* research consents from 21,000 parents and manually linking this with the corresponding samples arriving for routine screening was laborious for the NBS laboratory and overwhelming for all the maternity wards involved. A more streamlined approach will be required for the evaluation of further expansions of our routine NBS program.

We have not performed a formal cost effectiveness calcultation as this was not part of the main scope of the study. The presented NGS dependent algorithm has shown itself to be economically practical in full production. As costs for sequencing fall, new methods which also allow the detection of deep intronic variants, CNVs and other structural variants will be applicable to DBS samples (72, 73). The ethical concerns with this approach need to be addressed. Extensive *in silico* filtering to only include the relevant genes will be needed. Obligations to look for findings in other disease related genes or to report carrier status must also be avoided in order to prevent the multiple ethical dilemmas which could otherwise arise.

The utility and efficacy of 2nd tier NGS in SCID screening has been documented. The use of the original screening DBS sample has the following advantages:

- Molecular results within 2–3 working days
- Reduced need for a second DBS sample
- Reduced the total number of recalls, redraws and false positives
- Reduced need for pediatric consultations and flow cytometry to exclude false positives among the lower intermediate TRECs (5–20/μl)
- No unnecessary worries inflicted on the parents to newborns with TRECs 5–20/μl
- Ability to detect atypical and leaky SCIDs and other T cell deficiencies
- Shorter time from birth to confirmation of the specific immunodeficiency disorder.

The disadvantages

- Equipment cost and running cost of NGS analyses
- Inability to identify immunodeficiencies with intermediate low TREC values and variants in genes not included in the current/available gene panels
- Structural variants and intronic variants can be missed by exon based targeted NGS panels.

In conclusion, NGS integrated in the nationwide NBS algorithm rapidly delineates the specific molecular diagnosis and provides useful information for management, targeted therapy, and follow-up. Rapid detection may prevent breast milk transmitted CMV infection and other pre-HSCT acquired infections, optimizing pre-HSCT health condition. Meanwhile, a higher TREC cut-off threshold combined with NGS allows the detection of atypical, leaky SCIDs and other combined immunodeficiencies. This study forms a proof-of-concept for the integration of NGS methods into NBS laboratory routines and lays the foundations for the use of NGS methods in rapid confirmatory testing for further NBS conditions. There is still development work to be done, not least with regard to continuously updating and evaluating the list of genes included in panels; but the combination of speed, precision, cost and generalisability are strong arguments for adopting of this approach. While the overall cost of NBS seen in isolation is increased, it is important to note that the efficiency gains, improved management, and increased precision enabled by this screening approach contribute to a significant saving from a broader healthcare perspective.

## Data Availability Statement

All datasets generated for this study are included in the article/Supplementary Material.

## Ethics Statement

The studies involving human participants were reviewed and approved by Regional Committees for Medical and Health Research Ethics South East Norway. Written informed consent to participate in this study was provided by the participants' legal guardian/next of kin. Written informed consent was obtained from the minor(s)' legal guardian/next of kin for the publication of any potentially identifiable images or data included in this article.

## Author Contributions

JS: designed the pilot, responsible for establishing all SCID screening laboratory activities including TREC test, and NGS analyses. KG: started the TREC testing and prior to the pilot. EL, MB, AT, LS, MY-A, SH, RH, and HG: TREC testing, NGS analyses and interpretation of the gene variants. HE and TØ: responsible for treatment of all the SCID patients in Norway, research partners. TA: former PI of the NBS for SCID project, initiated TREC project and pilot. LO: immunological laboratory investigations. GK: CMV testing. HS: WES tests and gene variant evaluation. LM: introduced the SCID screening to the clinic. ADR: bioinformatics and providing data sets. TT and JJ: responsible for the algorithm and medical follow-up in the newborn screening. AL and AER: NICU patients. CA and JB: responsible for the bone marrow transplantations. TR: head of division, supported the pilot, involved in the project design and applied for nationwide SCID screening. RP: head of department, responsible for the newborn screening unit, and initiated the projects including nationwide SCID screening in Norway. AS-P: PI of the NBS for SCID project and the retrospective studies, designed the NBS gene panels and responsible for the gene variant evaluations. AS-P wrote the manuscript together with JS and KG. All authors contributed to the article and approved the submitted version.

## Conflict of Interest

The authors declare that the research was conducted in the absence of any commercial or financial relationships that could be construed as a potential conflict of interest.

## Footnotes

^1^https://primaryimmune.org/idf-advocacy-center/idf-scid-newborn-screening-campaign

^2^https://www.hrsa.gov/advisory-committees/heritable-disorders/rusp/index.html

^3^https://www.g-ba.de/beschluesse/3586/

^4^Clinical and Laboratory Standards Institute (CLSI). Newborn blood spot screening for severe combined immunodeficiency by measurement of T-cell receptor excision circles; Approved guideline. CLSI NBS06-A: 1ED 2013.

## Supplementary Material

Supplemental Figure S1 **(A)** TREC values related to birth weight in all the 21,232 individuals included in the pilot study. **(B)** TREC values related to gestational age in all the 21,232 individuals included in the pilot study, plus the six individuals referred with severe primary immunodeficiencies, born in the same time period as the pilot, but at other hospitals.

Supplemental Figure S2 **(A)** TREC values related to gender in 14,000 individuals (6,688 females and 7,250 males) included in the pilot study. **(B)** Birth weight related to gender in 14,000 individuals (6,688 females and 7,250 males) included in the pilot study.

Supplemental Table S1 New SCIDs and severe T cell deficiencies detected in the pilot project.

Supplemental Table S2 The individuals with the lowest TREC values in the pilot project.

Supplemental Table S3 Individuals with PIDs born outside pilot test region.

Supplemental Table S4 Retrospective study: TRECs and NGS on DNA from the original NBS DBS in individuals with known SCID or severe T cell deficiency.

Supplemental Table S5 New SCIDs and severe PIDs identified during the national newborn screening.

Supplemental Table S6 The individuals with the lowest TREC values on the national screening 2018-2019.

Supplemental Table S7 Newborn screening gene panels, versions 1-4.
