# Supplementary figures and images for: Second-Tier Next Generation Sequencing Integrated in Nationwide Newborn Screening Provides Rapid Molecular Diagnostics of Severe Combined Immunodeficiency
Source: Front Immunol. 2020 Jul 9;11:1417. doi: 10.3389/fimmu.2020.01417 (PMC7381310; doi:10.3389/fimmu.2020.01417)

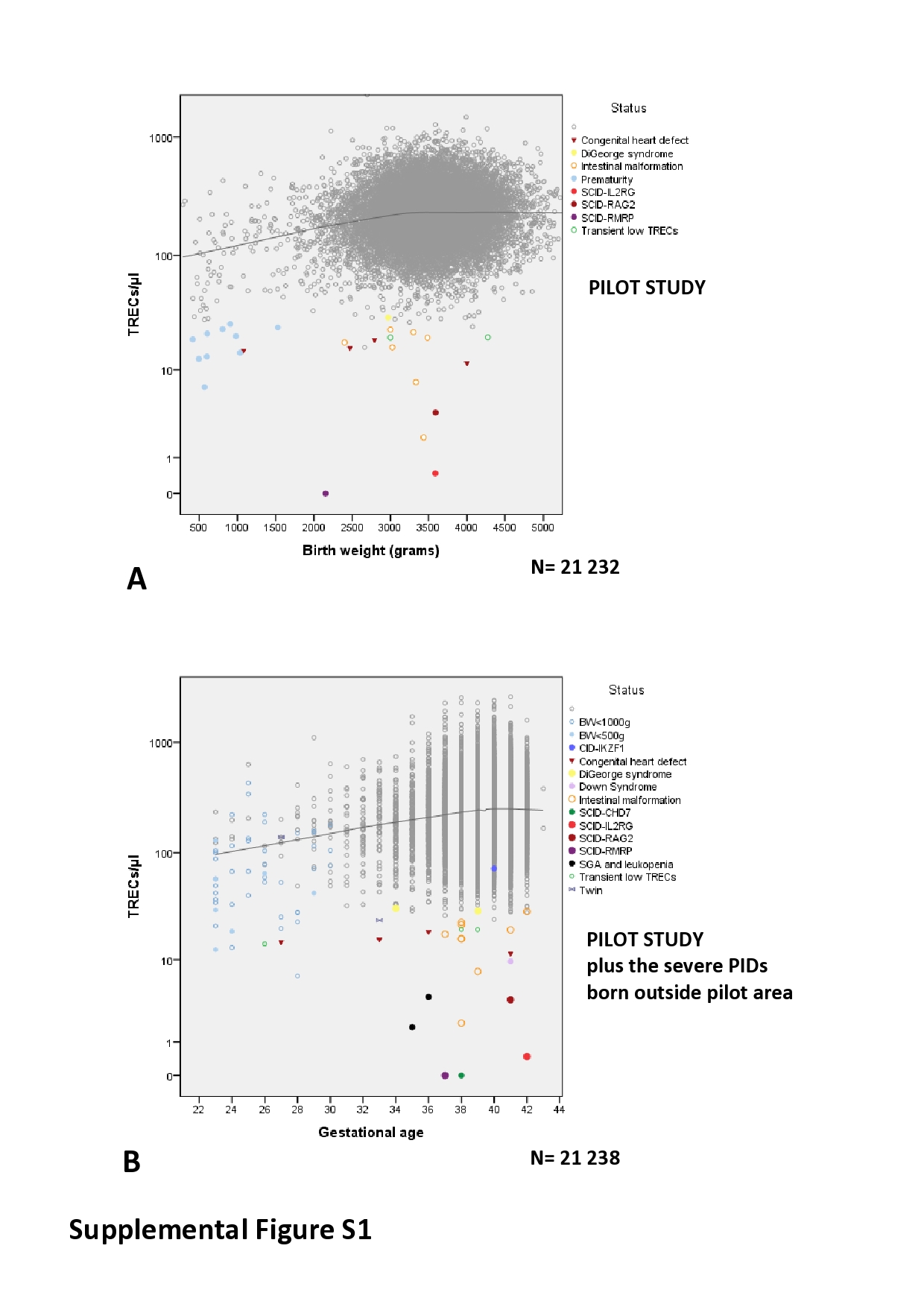

Supplement: Supplemental Figure S1 — (A) TREC values related to birth weight in all the 21,232 individuals included in the pilot study. (B) TREC values related to gestational age in all the 21,232 individuals included in the pilot study, plus the six individuals referred with severe primary immunodeficiencies, born in the same time period as the pilot, but at other hospitals. [file Image_1.jpeg]

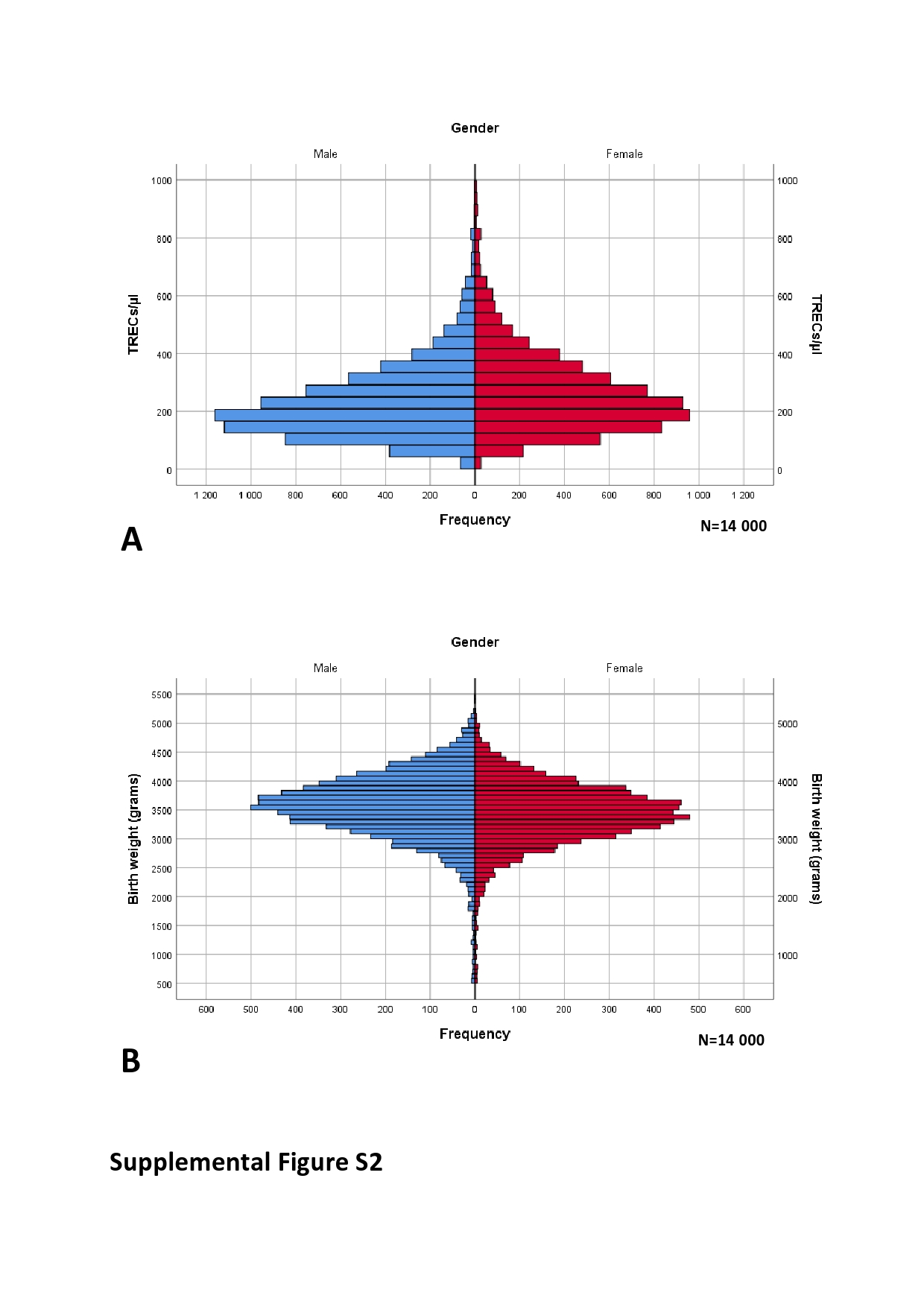

Supplement: Supplemental Figure S2 — (A) TREC values related to gender in 14,000 individuals (6,688 females and 7,250 males) included in the pilot study. (B) Birth weight related to gender in 14,000 individuals (6,688 females and 7,250 males) included in the pilot study. [file Image_2.jpeg]
