# Supplementary material for: Second-Tier Next Generation Sequencing Integrated in Nationwide Newborn Screening Provides Rapid Molecular Diagnostics of Severe Combined Immunodeficiency
Source: Front Immunol. 2020 Jul 9;11:1417. doi: 10.3389/fimmu.2020.01417 (PMC7381310; doi:10.3389/fimmu.2020.01417)
Supplement: Supplemental Table S1 — New SCIDs and severe T cell deficiencies detected in the pilot project. [file Table_1.pdf]

Supplemental Table S1 | New SCIDs and severe T cell deficiencies detected in the pilot project.

Patient ID	SCID_1	SCID_2	SCID_3	Age matched references
Symptoms at time of diagnosis	Healthy	Healthy	Skeletal dysplasia, total Hirschsprung	
Gender	Male	Male	Female	
GA w	42	41	37	
BW g	3592	3588	2152	
TRECs/µl Initial NBS	0.48	3.8 - 18.2 - 7.2	0	
DBS sample collected	Day 3	Day 3	Day 3	
Received NBS lab	Day 4	Day 6	Day 3	
TREC tested	Day 15/16	Day 12/13	Day 11	
TREC results available	Day 16	Day 12	Day 11	
Repeated TRECs/ul (age)	0 (Day 19)	9.1 (Day 17)<br>7.6 (9 months)	ND	
1st FACS (age):	FACS (Days 19 and 21):	FACS (Days 17 and 24):	1st FACS: (day 10)	
CD3 x10^6/L	58-29	1591-1500	198	2300-7000 x10^6/L
CD4 x10^6/L	<5	1303-1215	109	1700-5300 x10^6/L
CD8 x10^6/L	27-14	268-262	46	400-1700 x10^6/L
CD19 x10^6/L	510-611	16	ND	600-1900 x10^6/L
NK x10^6/L	169-41	903-489	ND	200-1400 x10^6/L
CD4+ naïve %	0%	77-69	ND	66-100%
RTE % of naïve CD4+	0%	56-50	ND	50-100%
Lymphocytes x10^9/L	0.8-0.7 (Days 18-21)	2.6-2.5-2.2 (Days 17-19-24)	ND	2.0-17 x10^9/L
WBC x10^9/L	5.9-4.8 (Days 18-21)	6.4-5.9-4.3 (Days 17-19-24)	5.2	5.0-21 x10^9/L
Age at molecular diagnosis	Day 22	Day 17	After death	
Gene	*IL2RG*	*RAG2*	*RMRP*	
SNV/CNV	c.[359dupA];[0]<br>HEMI	c.[1367C>T];[1367C>T]<br>HOM	n.[71A>G];[71A>G]<br>HOM	
Predicted effect on protein	p.Glu121Glyfs*47	p.Ala456Val	NA	
Refseq	NM_000206.2	NM_000536.3	NR_003051.3	
Classification ACMG[1]	5	3	5	
HGMD/ClinVar	CI972629	Not, but p.Ala456Thr reported in HGMD: CM0910583	CR011576/<br>RCV000015275.24,<br>SCV000035534	
gnomAD allele frequency	0	0	0.001,<br>no homozygotes	
Panel	PIDv2	PIDv2	PIDv2	
Mean X reads	242	556	305	
>100X	85%	92%	85%	
>20X	95%	96%	95%	
Other gene tests	Sanger validation	Sanger validation	Sanger validation	
Disease development, Treatment and outcome	Successful HSCT 3 months of age	Successful HSCT 14 months old. Atopic eczema, no skin GVHD. Hemolytic anemia at 21 months, resolved with Rituximab /steroid treatment.	Deceased 1 month old due to severe intestinal abnormality with complete Hirschsprungs disease	
Donor type<br>Preconditioning:<br>GVHD prophylaxis:	9/10 MUD,<br>Treo, Flu, ATG<br>ATG, CyA, MMF	10/10 MUD,<br>Treo, Flu, ATG<br>ATG, CyA, MMF		
CMV on initial card	Negative	Negative	NA	
Breastfeeding stopped	Day 17	Day 16	NA	
CMV serology mom	Negative, day 25	IgG positive, IgM negative, day 20	NA	
CMV infected	NO	NO	NA	

Latest FACS (time)q	FACS (1y post-transplant):	FACS (1y post-transplant):	NA	
CD3 x10^6/L	4597	3020	NA	1400-8000 x10^6/L
CD4 x10^6/L	3228	1409	NA	900-5500 x10^6/L
CD8 x10^6/L	1196	1414	NA	400-2300 x10^6/L
CD19 x10^6/L	1424	805	NA	600-2700 x 10^6/L
NK x10^6/L	216	469	NA	100-1400 x 10^6/L
Naïve CD4+%	85.2	ND	NA	
RTE %	91.1	ND	NA	
Lymphocytes x10^9/L	5.6 - 6.1	2.0	NA	2.0-11 x 10^9/L
WBC x10^9/L	8.6	5.7	NA	6.0-17 x 10^9/L
IgG g/L	9.9	6.7	NA	5.0-11 g/L
IgA g/L	< 0.10	1.6	NA	0.1-1.0 g/L
IgM g/L	1.9	0.48	NA	0.3-1.6 g/L
Parental ethnicity	Norwegian	Estonia	Somalia	

Abbreviations:
ACMG, American College of Medical Genetics and Genomics' Guidelines for variant interpretation and classification; ATG, Antithymocyte globulin; BW, Birth weight; CNV, Copy number variation; CyA, Cyclosporin A (Sandimmun); DBS, Dried blood spot; FACS, fluorescence-activated cell sorting /lymphocyte flow cytometry; Flu, Fludarabine; g, gram; GA, Gestational age; gnomAD, The Genome Aggregation Database; GVHD, Graft-versus-host disease; HGMD, The Human Gene Mutation Database; HGNC, The HUGO Gene Nomenclature Committee; HSCT, Hematopoietic Stem Cell Transplantation; MMF, Mycophenolate Mofetil (CellCept®); MUD, Matched unrelated donor; NA, Not applicable; NBS, Newborn screening; ND, Not done; PIDv2, Primary immunodeficiency research panel version 2; RefSeq, The National Center for Biotechnology Information Reference Database; RTE, Recent thymic emigrants (CD4+, CD45RA+, CD31+); SCID, Severe combined immunodeficiency; SNV, Single nucleotide variant; TREC, T-cell receptor excision circles; Treo, Treosulfan; y, year; w, week; WBC, white blood cells

Resources:

ClinVar, https://www.ncbi.nlm.nih.gov/clinvar/
Gene names according to HGNC, https://www.genenames.org/
Gene variant classification according to ACMG standards and guidelines, Genetics in Medicine, 2015[1]
gnomAD, https://gnomad.broadinstitute.org

Reference:

1. Richards, S., Aziz, N., Bale, S., Bick, D., Das, S., Gastier-Foster, J., Grody, W.W., Hegde, M., Lyon, E., Spector, E., et al. (2015). Standards and guidelines for the interpretation of sequence variants: a joint consensus recommendation of the American College of Medical Genetics and Genomics and the Association for Molecular Pathology. Genet Med 17, 405-424.
