# Supplementary material for: Second-Tier Next Generation Sequencing Integrated in Nationwide Newborn Screening Provides Rapid Molecular Diagnostics of Severe Combined Immunodeficiency
Source: Front Immunol. 2020 Jul 9;11:1417. doi: 10.3389/fimmu.2020.01417 (PMC7381310; doi:10.3389/fimmu.2020.01417)
Supplement: Supplemental Table S2 — The individuals with the lowest TREC values in the pilot project. [file Table_2.pdf]

Supplemental Table S2 | The individuals with the lowest TREC values in the pilot project.

TRECs/µl				GA	NICU	Hospital	PIDv2 gene panel	Known condition at birth	Reported and/or redraw requested	Findings and history
**Initial**	**Re-run**	**New extraction**	**Mean**							
0	0	0	0	37	Yes	OUS	Yes	Intestinal malformation and skeletal dysplasia	Reported	*RMRP*-SCID, Deceased
0.48	0	0	0.16	42	No	Other	Yes	None	Reported	*IL2RG*-SCID, Transplanted
1.96	9.2	17.7	9.6	38	Yes	OUS	Yes	Intestinal malformation and pulmonal hypertension	Redraw requested	No pathogenic variants detected, TRECs normalizing
3.8	18.2	7.2	9.7	41	No	Other	Yes	None	Reported	*RAG2*-SCID, Transplanted
6.8	18.1	5.3	8.6	28	Yes	OUS	Yes	Prematurity, <1000g	Redraw requested	No pathogenic variants detected
7.6	41.5	22.1	23.7	39	Yes	OUS	No	Intestinal malformation	Reported	
11.5	20.2	11.8	14.5	41	Yes	OUS	Yes	Congenital heart disease	Redraw requested	No pathogenic variants detected
12.6	25.2	13.9	11.3	23	Yes	OUS	No	Prematurity, <500g	Not reported	
13.2	3.5	5.6	7.4	24	Yes	OUS	Yes	Prematurity, <1000g	Not reported	No pathogenic variants detected
14.3	23.7	16.2	18	26	Yes	OUS	No	Prematurity, 1000g	Redraw requested	TRECs normalizing
14.9	27.1	16.2	19.4	27	Yes	OUS	No	Prematurity, 1000g, cystic hygroma, polyhydramnios and congenital heart disease	Redraw requested	Deceased 2 weeks old, *RIT1*-cardiomyopathy, identified by clinical WES
15.8	35.8	13.5	21.7	33	Yes	OUS	No	Congenital heart disease	Redraw requested	TRECs normalizing
16	10.9	1	9.3	38	Yes	OUS	No	Intestinal malformation, diaphragmatic hernia and tracheomalacia	Redraw requested	TRECs normalizing
17.7	17.8	13	16.2	37	Yes	OUS	Yes	Intestinal malformation, gastroschisis	Not reported	No pathogenic variants detected
18.6	19.2	7.8	15.2	36	Yes	OUS	No	Congenital heart disease	Redraw requested	TRECs normalizing
18.8	4.1	10.3	11.1	24	Yes	OUS	No	Prematurity, <500g	Redraw requested, but not received	
19.4	34.3	37.4	30.4	41	Yes	OUS	No	Intestinal malformation	Not reported	
19.5	23.3	14.2	19	39	Yes	OUS	No	Birth asphyxia and meconium aspiration	Redraw requested	TRECs normalizing
19.6	16.6	35.4	23.9	38	No	Other	Yes	Neonatal reduced health condition	Not reported, but redraw requested later	No pathogenic variants detected, TRECs normalizing
20.1	27.8	22.7	23.5	27	Yes	OUS	No	Prematurity, <1000g, and lung disease	Not reported	His monozygous twin had normal TREC levels
21.2	-	-	21.2	23	Yes	OUS	No	Prematurity, <1000g, Necrotizing enterocolitis	Not reported	Deceased 4 weeks old
22.1	32.6	29.2	28	39	Yes	OUS	No	Congenital heart disease	Not reported	DiGeorge syndrome, identified by clinical MLPA
22.9	12.7	32	22.5	38	Yes	OUS	No	Intestinal malformation and congenital heart disease	Redraw requested	TRECs normalizing
23.1	36.2	12.5	24	28	Yes	OUS	No	Prematurity, <1000g	Not reported	

Abbreviations: g, gram; GA, Gestational age; HGNC, The HUGO Gene Nomenclature Committee; MLPA, Multiplex ligation-dependent probe amplification; NICU, Neonatal intensive care unit; OUS, Oslo University Hospital; PID, Primary immunodeficiency; SCID, Severe combined immunodeficiency; TREC, T-cell receptor excision circles; WES, whole exome sequencing

Resources: Gene names according to HGNC, https://www.genenames.org/; Gene variant nomenclature according to the HGVS recommendations[1], http://www.HGVS.org/varnomen
