# Supplementary material for: Second-Tier Next Generation Sequencing Integrated in Nationwide Newborn Screening Provides Rapid Molecular Diagnostics of Severe Combined Immunodeficiency
Source: Front Immunol. 2020 Jul 9;11:1417. doi: 10.3389/fimmu.2020.01417 (PMC7381310; doi:10.3389/fimmu.2020.01417)
Supplement: Supplemental Table S3 — Individuals with PIDs born outside pilot test region. [file Table_3.pdf]

Supplemental Table S3 | Individuals with PIDs born outside pilot test region. TRECs and PIDv2 panel testing on the original DBS and on new samples.

Patient ID	PID_1	PID_2	PID_3	PID_4		PID_5		PID_6
Year	2016	2016	2017	2017		2017		2017
Symptoms at time of diagnosis	SGA, microcephaly,<br>transient lymphopenia,<br>persistent neutropenia and<br>low number of platelets	SGA, microcephaly,<br>failure to thrive,<br>pigment patches skin	Heart defect,<br>choanal atresia,<br>coloboma, infections	Heart defect,<br>prematurity,<br>Downs syndrome		Heart defect,<br>dysmorphic features		Infections,<br>failure to thrive
Gender	Female	Male	Male	Male		Male		Female
GA w	35	36	36	33		34		40
BW g	1920	1534	2445	1844		1570		3430
TRECs/µl at birth	4.69	1.9	0	9.7		15.7		71.8
Repeated TRECs/ul (age)	23.8 (3 months)<br>0 (12 months)	0 (4 months)	0 (1.5 months)	0 (2 months)<br>17-32 (2.5 months)		ND		12.2 (6.8 months)
1st FACS (age):	2.5 months	3 months	1.5 months	2 months	2.5 months	Day 1	3 months	6.8 months
CD3 x10^6/L	**918 (2300-6500)**	**98 (2300-6500) x10^6/L**	**617 (2300-7000) x10^6/L**	**212 (2300-6500) x10^6/L**	**1249 (2300-6500) x10^6/L**	**1146 (600-5000) x10^6/L**	**3922 (2300-6500) x10^6/L**	**1319 (2400-6900) x10^6/L**
CD4 x10^6/L	**190 (1500-5000) x10^6/L**	**90 (1500-5000) x10^6/L**	**552 (1700-5300) x10^6/L**	**149 (1500-5000) x10^6/L**	**890 (1500-5000) x10^6/L**	**673 (400-3500) x10^6/L**	2199 (1500-5000) x10^6/L	**918 (1400-5100) x10^6/L**
CD8 x10^6/L	**443 (500-1600) x10^6/L**	**9 (500-1600) x10^6/L**	**73 (400-1700) x10^6/L**	**60 (500-1600) x10^6/L**	**356 (500-1600) x10^6/L**	**465 (200-1900) x10^6/L**	1597 (500-1600) x10^6/L	**383 (600-2200) x10^6/L**
CD19 x10^6/L	3506 (600-3000) x10^6/L	**72 (600-3000) x10^6/L**	4067 (600-1900) x10^6/L	**256 (600-3000) x10^6/L**	**308 (600-3000) x10^6/L**	**269 (100-1900) x10^6/L**	3367 (600-3000) x10^6/L	**9 (700-2500) x10^6/L**
NK x10^6/L	477 (100-1300) x10^6/L	**7 (100-1300) x10^6/L**	4551 (200-1400) x10^6/L	**64 (100-1300) x10^6/L**	**117 (100-1300) x10^6/L**	**236 (100-1900) x10^6/L**	1928 (100-1300) x10^6/L	**40 (100-1000) x10^6/L**
Lymphocytes x10^9/L	7.2 (2-17) x10^9/L	**0.3 (2-17) x10^9/L**	NA (2-17) x10^9/L	0.6 (2-17) x10^9/L	NA (2-17) x10^9/L	1.6 (2-15) x10^9/L	6.1 (2-17) x10^9/L	**1 (3.5-13.5) x10^9/L**
Neutrophils x10^9/L	**0.4 (1-9) x10^9/L**	**1.0 (1-9) x10^9/L**	6.3 (1-9) x10^9/L	8.5 (1-9) x10^9/L	1.4 (1-9) x10^9/L	4.0 (3-25) x10^9/L	3.6 (1-9) x10^9/L	3.1 (1-8.5) x10^9/L
WBC x10^9/L	8.4 (5-19) x10^9/L	**2.1 (5-19) x10^9/L**	11.6 (5-19) x10^9/L	10.8-(5-19) x10^9/L	4.3 (5-19) x10^9/L	6.1 (9-30) x10^9/L	10.9 (5-19) x10^9/L	**4.4 (6-17) x10^9/L**
IgG g/L	**10.6 (3-7) g/L**	**2.4 (3-7) g**/L	4.3 (3-7) g/L	2.4 (3-7) g/L	NA	NA	3.3 (3-7) g/L	**< 1.0 (3-7) g/L**
IgA g/L	< 0.1 (0-0.3) g/L	< 0.2 (0-0.3) g/L	0.11 (0-0.3) g/L	0.4 (0-0.3) g/L	NA	NA	0.12 (0-0.3) g/L	< 0.1 (0-0.3) g/L
IgM g/L	0.22 (0.1-0.8) g/L	0.5 (0.1-0.8) g/L	0.44 (0.1-0.8) g/L	0.2 (0.1-0.8) g/L	NA	NA	0.77 (0.1-0.8) g/L	1.1 (0.1-0.8) g/L
Gene	unknown	unknown	*CHD7*	Trisomy 21		*TBX1*		*IKZF1*
SNV/CNV	NA	NA	c.[5833C>T];[=]	47,XY,+21		22q11.21 del		c.[476A<G];[=]
protein	NA	NA	p.Arg1945*	NA		NA		p.Asn159Ser
Refseq	NA	NA	NM_017780.3	NA		NA		NM_006060.4
Classification ACMG[1]	NA	NA	5	NA		NA		4
HGMD/ClinVar	NA	NA	RCV-000258138.1<br>CM060228	NA		NA		CM173423[2]
gnomAD allele frequency	NA	NA	0 in gnomAD	NA		NA		0 in gnomAD, but<br>one heterozygote<br>individual reported<br>among 60 000 in ExAC
Gene panel	PIDv2 and IEM	PIDv2 and Hematology	NBSv1	NBSv1		NBSv1		PIDv2
Other gene tests	mtDNA deletion test normal.<br>WES trio and WGS trio,<br>No pathogenic variants<br>detected	Fanconi anemia on<br>Mitomycin C test.<br>WES trio and WGS trio,<br>No pathogenic variants detected	Sanger validation	Trisomy test		MLPA		Sanger validation
Disease development	Short stature,<br>Microcephaly and DD/ID.<br>Developed diabetes mellitus,<br>but<br>Pearson syndrome excluded	DD/ID,<br>Microcephaly<br>OFC 44.3cm at 2y3m<br>= 3 cm below 2.5p<br>Short stature 86 cm at 2.5y, at 3p	Short stature,<br>Microcephaly, Seizures,<br>Tracheostomia, needs<br>respiratory support.	Heart surgery of ASD, VSD, PDA at 2 months				*P.jirovecii*

Treatment and outcome	Treated with immunomodulatory agents, steroids, tacrolimus. Deceased at 1.5 years of age after *P.jirovecii* infection followed by respiratory failure.	Successful HSCT 6,5 months old 10/10 MUD Preconditioning regimen: RIC Fanconi anemia protocol Flu, CP, ATG	At 2 years of age: prophylactic antibiotics, antifungal, antiviral and Sc immunoglobulins. Thymus transplantation were considered earlier	Lymphopenia 1.9x10^9/L at 1 years of age, but no recurrent infections	Prophylactic antibiotics	Successful HSCT[3] 9 months of age 11/12 MUD
Parental ethnicity	Norwegian	Norwegian	Arab	Norwegian	Pakistan	Norwegian

Abnormal laboratory results are marked in bold in the table above.

Abbreviations:
aCGH, array comparative genomic hybridization chromosomal/chromosomal microarray; ACMG, American College of Medical Genetics and Genomics' Guidelines for variant interpretation and classification; ATG, Antithymocyte globulin; BW, Birth weight; CNV, Copy number variation; CP, Cyclophosphamide; DD/ID, Developmental delay/Intellectual disability; FACS, fluorescence-activated cell sorting /lymphocyte flow cytometry; Flu, Fludarabine; g, gram; GA, Gestational age; gnomAD, The Genome Aggregation Database; Hematology, Ion Ampliseq™ Hematology research panel; HGMD, The Human Gene Mutation Database; HGNC, The HUGO Gene Nomenclature Committee; HSCT, Hematopoietic Stem Cell Transplantation; IEM, Ion Ampliseq™ Inborn Errors of Metabolism research panel; MLPA, multiplex ligation-dependent probe amplification; MUD, Matched unrelated donor; NA, Not applicable; NBS, Newborn screening; NBSv1, Newborn screening customized 160-gene panel version 1; ND, Not done; OFC, occipital frontal circumference; PIDv2, Primary immunodeficiency research panel version 2; PID, Primary immunodeficiency; Refseq, The National Center for Biotechnology Information Reference Database; RIC, Reduced intensity conditioning; SCID, Severe combined immunodeficiency; Sc, Subcutaneous; SGA, Small for gestational age; SNV, Single nucleotide variant; TREC, T-cell receptor excision circles; Trio-test; testing the child and both parents in comparison; WES, whole exome sequencing; WGS, whole genome sequencing; y, year; w, week; WBC, white blood cells

Resources:

ClinVar, https://www.ncbi.nlm.nih.gov/clinvar/
Gene names according to HGNC, https://www.genenames.org/
Gene variant classification according to ACMG standards and guidelines, Genetics in Medicine, 2015
gnomAD, https://gnomad.broadinstitute.org

References:

1. Richards, S., Aziz, N., Bale, S., Bick, D., Das, S., Gastier-Foster, J., Grody, W.W., Hegde, M., Lyon, E., Spector, E., et al. (2015). Standards and guidelines for the interpretation of sequence variants: a joint consensus recommendation of the American College of Medical Genetics and Genomics and the Association for Molecular Pathology. Genet Med 17, 405-424.

2. Yoshida, N., Sakaguchi, H., Muramatsu, H., Okuno, Y., Song, C., Dovat, S., Shimada, A., Ozeki, M., Ohnishi, H., Teramoto, T., et al. (2017). Germline IKAROS mutation associated with primary immunodeficiency that progressed to T-cell acute lymphoblastic leukemia. Leukemia 31, 1221-1223.

3. Kellner, E.S., Krupski, C., Kuehn, H.S., Rosenzweig, S.D., Yoshida, N., Kojima, S., Boutboul, D., Latour, S., Barlogis, V., Galambrun, C., et al. (2019). Allogeneic hematopoietic stem cell transplant outcomes for patients with dominant negative IKZF1/IKAROS mutations. J Allergy Clin Immunol 144, 339-342.
