# Supplementary material for: Second-Tier Next Generation Sequencing Integrated in Nationwide Newborn Screening Provides Rapid Molecular Diagnostics of Severe Combined Immunodeficiency
Source: Front Immunol. 2020 Jul 9;11:1417. doi: 10.3389/fimmu.2020.01417 (PMC7381310; doi:10.3389/fimmu.2020.01417)
Supplement: Supplemental Table S4 — Retrospective study: TRECs and NGS on DNA from the original NBS DBS in individuals with known SCID or severe T cell deficiency. [file Table_4.pdf]

Supplemental Table S4 | Retrospective study: TRECs and NGS on DNA from the original NBS DBS in individuals with known SCID or PIDs.

	**INDIVIDUALS WITH KNOWN SCID or severe T-cell deficiency**									**OTHER KNOWN PIDs**	
Patient ID	KID_1	KID_2	KID_3	KID_4	KID_5	KID_6	KID_7	KID_8	KID_9	KID_10	KID_11
Year	2012	2010	2010	2015	2012	2014	2015	2006	2009	2009	2012
Gender	Male	Female	Female	Female	Female	Male	Male	Female	Male	Male	Female
GA	37	34	38,5	42	40	42	39	38	39	40	38
BW	2954	1999	2618	3360	4135	4420	2484	NA	3445	3775	2950
TRECs/µl Initial NBS	0	0	0	0	11	0	0	0	0	25.5	60
Gene	*IL2RG*	*LIG4*	*IL7R*	*ADA*	*PGM3*	*JAK3*	*TBX1*	*TBX1*	*TBX1*	*IKZF1*	*RECQL4*
SNV/CNV	c.[924+5G>A] ;[0]	c.[1341G>T] ;[482delC]	c.[707-2A>G] ;[707-2A>G]	c.[7C>T] ;[7C>T]	c.[737A>G]; [737A>G]	c.1837C>T; c.1695C>A	22q11.21 del	22q11.21 del	22q11.21 del	c.1618388_ 589+2308del (16.8 kb del exons 4-5)	c.[2269]C>T; ND
Zygosity	HEMI	COMP HET	HOM	HOM	HOM	COMP HET	HET	HET	HET	HET	COMP HET
Effect on protein	Splice defect	p.Trp447Cys; p.Ala161Valfs*6	Splice defect	p.Gln3*	p.Ala246Gly	p.Arg613*; p.Cys565*	loss	loss	loss	Inframe deletion	p.Gln757*
Refseq	NM_ 000206.2	NM_ 002312.3	NM_ 002185.3	NM_ 000022.2	NM_ 015599.2	NM_ 000215.3	NM_ 080647.1	NM_ 080647.1	NM_ 080647.1	NM_ 006060.5	NM_ 004260.3
ACMG[1]		4;4		5	5	5;5					5
HGMD/ ClinVar		CM157470; CD171521		CM950010, RCV000433743.1		CM1516563; CM950705, RCV000009957.4					RCV000006435.4[2] Immunodeficiency and *RECQL4*[3]
gnomAD		0.00001219; 0		0 in gnomAD but known to be recurrent in Somali population		0.00001219; 0					0.0001193
Panel	PIDv1	PIDv1	PIDv1	PIDv2	PIDv1	PIDv1	PIDv2	PIDv2	PIDv2	PIDv2	PIDv1
Mean X reads	313	235	310	304	358	377	120	292	182		245
>100X	84%	75%	82%	87%	84%	91%	51%	75%	66%		84%
>20X	96%	95%	96%	97%	97%	98%	93%	95%	94%		97%
Patient IDs in other reports	116.1 in JACI 2017[4]	38.1 in JACI 2017[4]	114.1 in JACI 2017[4]		110.1 in JACI 2017[4], P1 in AJHG 2014[5]	117.1 in JACI 2017[4]	Deceased, Thymus transplanted	Deceased	Deceased	E2 in NEJM 2016[6]	127.1 in JACI 2017[4]

Abbreviations:

ACMG, American College of Medical Genetics and Genomics' Guidelines for variant interpretation and classification; BW, Birth weight; CNV, Copy number variation; COMP HET, Compound heterozygote; g, gram; GA, Gestational age; gnomAD, The Genome Aggregation Database; HGMD, The Human Gene Mutation Database; HGNC, The HUGO Gene Nomenclature Committee; HOM, Homozygote; ND, Not detected; PIDs, Primary immunodeficiencies; PIDv2, Primary immunodeficiency research panel version 2; RefSeq, The National Center for Biotechnology Information Reference Database; SCID, Severe combined immunodeficiency; SNV, Single nucleotide variant; TREC, T-cell receptor excision circles; w, weeks

Resources:

ClinVar, https://www.ncbi.nlm.nih.gov/clinvar/
Gene names according to HGNC, https://www.genenames.org/
Gene variant evaluation according to ACMG standards and guidelines, Genetics in Medicine, 2015[1]
gnomAD, https://gnomad.broadinstitute.org

References:

1. Richards, S., Aziz, N., Bale, S., Bick, D., Das, S., Gastier-Foster, J., Grody, W.W., Hegde, M., Lyon, E., Spector, E., et al. (2015). Standards and guidelines for the interpretation of sequence variants: a joint consensus recommendation of the American College of Medical Genetics and Genomics and the Association for Molecular Pathology. Genet Med 17, 405-424.

2. Siitonen, H.A., Sotkasiira, J., Biervliet, M., Benmansour, A., Capri, Y., Cormier-Daire, V., Crandall, B., Hannula-Jouppi, K., Hennekam, R., Herzog, D., et al. (2009). The mutation spectrum in RECQL4 diseases. Eur J Hum Genet 17, 151-158.

3. Broom, M.A., Wang, L.L., Otta, S.K., Knutsen, A.P., Siegfried, E., Batanian, J.R., Kelly, M.E., and Shah, M. (2006). Successful umbilical cord blood stem cell transplantation in a patient with Rothmund-Thomson syndrome and combined immunodeficiency. Clin Genet 69, 337-343.

4. Stray-Pedersen, A., Sorte, H.S., Samarakoon, P., Gambin, T., Chinn, I.K., Coban Akdemir, Z.H., Erichsen, H.C., Forbes, L.R., Gu, S., Yuan, B., et al. (2017). Primary immunodeficiency diseases: Genomic approaches delineate heterogeneous Mendelian disorders. J Allergy Clin Immunol 139, 232-245.

5. Stray-Pedersen, A., Backe, P.H., Sorte, H.S., Morkrid, L., Chokshi, N.Y., Erichsen, H.C., Gambin, T., Elgstoen, K.B., Bjoras, M., Wlodarski, M.W., et al. (2014). PGM3 mutations cause a congenital disorder of glycosylation with severe immunodeficiency and skeletal dysplasia. Am J Hum Genet 95, 96-107.

6. Kuehn, H.S., Boisson, B., Cunningham-Rundles, C., Reichenbach, J., Stray-Pedersen, A., Gelfand, E.W., Maffucci, P., Pierce, K.R., Abbott, J.K., Voelkerding, K.V., et al. (2016). Loss of B Cells in Patients with Heterozygous Mutations in IKAROS. N Engl J Med 374, 1032-1043.
