# Supplementary material for: Second-Tier Next Generation Sequencing Integrated in Nationwide Newborn Screening Provides Rapid Molecular Diagnostics of Severe Combined Immunodeficiency
Source: Front Immunol. 2020 Jul 9;11:1417. doi: 10.3389/fimmu.2020.01417 (PMC7381310; doi:10.3389/fimmu.2020.01417)
Supplement: Supplemental Table S5 — New SCIDs and severe PIDs identified during the national newborn screening. [file Table_5.pdf]

Supplemental Table S5 | New SCIDs and severe PIDs identified during the national newborn screening period 2018-2019

Patient ID	**SCID_4**	**SCID_5**		**SCID_6**	**SCID_7**	**CID_1**	Age matched references
Year	**2018**	**2018**		**2019**	**2019**	**2019**	
Symptoms at time of diagnosis	**Apparently healthy**	**Healthy**		**Microcephaly OFC 29cm, BL 43cm, left hydronephrosis**	**Healthy**	**Apparently healthy**	
Gender	**Male**	**Male**		**Female**	**Male**	**Female**	
GA w	**36**	**39**		**40**	**41**	**41**	
BW g	**3618**	**2515**		**2855**	**3540**	**4065**	
TRECs/µl Initial NBS (age)	**0 - 0**	**0.0 - 5.3 – 0.0**		**0 - 0 - 3.7**	**0 - 0 - 0**	**8.3 - 15.3 - 10.3**	
DBS sample collected	**Day 2**	**Day 2**		**Day 2**	**Day 2**	**Day 2**	
Received NBS lab	**Day 2**	**Day 8**		**Day 3**	**Day 3**	**Day 6**	
TREC tested	**Day 3/4**	**Day 12/13**		**Day 6**	**Day 3**	**Day 8**	
TREC results available	**Day 4**	**Day 13**		**Day 6**	**Day 4**	**Day 8**	
Repeated TRECs/ul (age)	**0 (Day 8)**	**0 (Day 14)**		**11 (Day 7)**	**0 (Day 5)**	**6.6-17.5 (Day 12)**	
1st FACS (age):	1st FACS (Day 8)	1st FACS		1st FACS (day 7)	1st FACS (Day 5)	FACS days 12 and 13	
CD3 x10^6/L	<5	3		725	<5	1588-1412	2300-7000 x10^6/L
CD4 x10^6/L	<5	1		538	<5	1285-1156	1700-5300 x10^6/L
CD8 x10^6/L	<5	1		177	<5	300-251	400-1700 x10^6/L
CD19 x10^6/L	<5	427		24	1518	301-313	600-1900 x10^6/L
NK x10^6/L	896	4		1172	<5	667-870	200-1400 x10^6/L
Lymphocytes x10^9/L	1.0	0.8		1.7	1.3	2.2	2.0-17 x10^9/L
WBC x10^9/L	6.6	4.6		5.7	15.3	8.2	5.0-21 x10^9/L
Age at molecular diagnosis	**Day 8**	**Day 15**		**Day 8**	**Day 6**	**No molecular diagnosis, Day 13 Ultrasound: thymus aplasia**	
Gene	***DCLRE1C Artemis***	***JAK3***		***NBN***	***IL2RG***	**None (day 9)**	
SNV/CNV	**c.[82C>G] HOM**	**c.[1767C>T]**	**c. [2077C>A]**	**c.[657_661delACAAA] HOM**	**c.[371T>C];[0]**	NA	
Predicted effect on protein	**p.Ala28Pro**	**Splice defect**	**p.Pro693Thr**	**p.Lys219Asnfs*16**	**p.Leu124Pro**	NA	
Refseq	NM_001033855.2	NM_000215.3	NM_000215.3	NM_002485,4	NM_000206.2	NA	
Classification ACMG	5	5	3	5	5	NA	
HGMD/ClinVar, Ref	CM101617,[1; 2]	CS971783/ RCV000256129.1,[3]	NA	CD982819/ RCV000007353.13,[4]	CM108804,[5]	NA	
gnomAD allele frequency	1/250956, 4×10^{-6} no homozygotes	1/171516, 6×10^{-6} no homozygotes	0	57/282132, 2×10^{-4} no homozygotes	0	NA	
Panel	**NBSv2**	**NBSv2**	**NBSv2**	**NBSv2**	**NBSv2**	**NBSv2 + PIDv2**	
Other gene tests	**Sanger validation**	**Sanger validation**	**Sanger validation**	**Sanger validation, other lab**	**Sanger validation**	**WES trio, aCGH**	
**Treatment**	**Successful HSCT**	**Successful HSCT**		**HSCT planned**	**HSCT planned**	**Clinical follow-up only**	

Disease development, Treatment and outcome	HSCT 3 months of age with modified preconditioning for RS-SCID Haploidentical donor, GVHD skin +14, SoluMedrol treated, resolved	HSCT 3 months of age with HLA matched CMV+ unrelated donor. Reactivation CMV 2m post-HSCT, see below	Profylaxis prior to HSCT: Iv Ig Panzyga 2.5g Hizentra 5ml sc /w since 6m age Bactrim 2,5 ml x 2 Diflucan 20mg x 1 Acyclovir/Zovirax 100mgx3	Profylaxis since day 9: Iv Ig Panzyga 2.5g Bactrim 2,5 ml x 1 at 6w Diflucan 20mg x 1 Acyclovir 100mgx3	Close clinical follow-up, thymus transplantation have been considered	
CMV on initial card	**Negative**	**Negative**	NOT TESTED	NOT TESTED	NOT TESTED	
Breastfeeding stopped	**Day 5**	**Day 14**	**Day 6**	**Day 4**	**Day 8**	
CMV serology mom (days after delivery)	**IgG positive, IgM negative, Results Day 9**	**IgG positive IgM negative, Results Day 17**	**IgG positive, IgM negative (mom tested 2 m prior to delivery)**	**IgG positive IgM negative Results Day 5,**	**IgG positive IgM negative Day 12**	
CMV infected	**NO**	**YES**	**NO**	**NO**	**NO**	
CMV treatment and further testing	NA	Day 14 sample: CMV 1036 IU/mL in plasma, results available Day 22<br>Day 23: changed from Aciclovir prophylaxis to Ganciclovir treatment with Valcyte® 16mg/kg x 2<br>2m post-HSCT: Reactivation with CMV 132 588 IU/mL in plasma, restarted Ganciclovir.<br>Later changed to Foscarnet due to resistance (UL97 M460MI, H520HQ)<br>Never had symptoms of infection, no CMV organ involvement	NA	NA	NA	
Latest FACS (time)	FACS (9m post-transplant):	FACS (6m post-transplant):	6m old	NA	4m old	
CD3 x10^6/L	2494	1052	346	NA	852	1400-8000 x10^6/L
CD4 x10^6/L	1329	488	295	NA	678	900-5500 x10^6/L
CD8 x10^6/L	802	506	54	NA	158	400-2300 x10^6/L
CD19 x10^6/L	242	741	53	NA	443	600-2700 x 10^6/L
NK x10^6/L	186	625	689	NA	254	100-1400 x 10^6/L
Lymphocytes x10^9/L	2.8	NA	0.7	NA	NA	2.0-11 x 10^9/L
WBC x10^9/L	7.0	NA	2	NA	NA	6.0-17 x 10^9/L
IgG g/L	6.2	NA	8.3 (on IgG treatment)	NA	NA	5.0-11 g/L
IgA g/L	< 0.10	NA	< 0.1	NA	NA	0.1-1.0 g/L
IgM g/L	0.96	NA	3	NA	NA	0.3-1.6 g/L
		6m post-HSCT, CMV < 36 IU/mL	6 m old, CMV <36 IU/mL			
Parental ethnicity	Turkish	Norwegian	Polish	Norwegian/Lithuanian	Norwegian	

Abbreviations: aCGH, array comparative genomic hybridization chromosomal/chromosomal microarray; ACMG, American College of Medical Genetics and Genomics' Guidelines for variant interpretation and classification; BW, Birth weight; CMV, *cytomegalovirus*; CNV, Copy number variation; DBS, Dry blood spot; FACS, fluorescence-activated cell sorting /lymphocyte flow cytometry, g, gram; GA, Gestational age; gnomAD, The Genome Aggregation Database; GVHD, Graft-versus-host disease; HGMD, The Human Gene Mutation Database; HGNC, The HUGO Gene Nomenclature Committee; HOM, Homozygous; HSCT, Hematopoietic Stem Cell Transplantation; m, Month; NA, Not applicable; NBS, Newborn screening; NBSv2, Newborn screening gene panel version 2; PIDv2, Primary immunodeficiency research panel version 2; PID, Primary immunodeficiency; RefSeq, The National Center for Biotechnology Information Reference Database; RS-SCID, Radiosensitive SCID;

SCID, Severe combined immunodeficiency; SNV, Single nucleotide variant; TREC, T-cell receptor excision circles; Trio-test; testing the child and both parents in comparison; WES, whole exome sequencing; w, week; WBC, white blood cells

Resources:
ClinVar, https://www.ncbi.nlm.nih.gov/clinvar/
Gene names according to HGNC, https://www.genenames.org/
Gene variant nomenclature according to the HGVS recommendations[6], http://www.HGVS.org/varnomen
Gene variant classification according to ACMG standards and guidelines, Genetics in Medicine, 2015[7]
gnomAD, https://gnomad.broadinstitute.org

References
DCLRE1C, c.[82C>G]: Pannicke et al Hum Mutat, 2010[1], Felgentreff et al JACI, 2015[2]
JAK3 c.[1767C>T]: Candotti et al Blood, 1997[3]
NBN c.[657_661delACAAA]: Varon et al Cell, 1998[4]
IL2RG c.[371T>C]: Lee et al JCI, 2010[5]

1. Pannicke, U., Honig, M., Schulze, I., Rohr, J., Heinz, G.A., Braun, S., Janz, I., Rump, E.M., Seidel, M.G., Matthes-Martin, S., et al. (2010). The most frequent DCLRE1C (ARTEMIS) mutations are based on homologous recombination events. Hum Mutat 31, 197-207.
2. Felgentreff, K., Lee, Y.N., Frugoni, F., Du, L., van der Burg, M., Giliani, S., Tezcan, I., Reisli, I., Mejstrikova, E., de Villartay, J.P., et al. (2015). Functional analysis of naturally occurring DCLRE1C mutations and correlation with the clinical phenotype of ARTEMIS deficiency. J Allergy Clin Immunol 136, 140-150 e147.
3. Candotti, F., Oakes, S.A., Johnston, J.A., Giliani, S., Schumacher, R.F., Mella, P., Fiorini, M., Ugazio, A.G., Badolato, R., Notarangelo, L.D., et al. (1997). Structural and functional basis for JAK3-deficient severe combined immunodeficiency. Blood 90, 3996-4003.
4. Varon, R., Vissinga, C., Platzer, M., Cerosaletti, K.M., Chrzanowska, K.H., Saar, K., Beckmann, G., Seemanova, E., Cooper, P.R., Nowak, N.J., et al. (1998). Nibrin, a novel DNA double-strand break repair protein, is mutated in Nijmegen breakage syndrome. Cell 93, 467-476.
5. Lee, P.P., Chan, K.W., Chen, T.X., Jiang, L.P., Wang, X.C., Zeng, H.S., Chen, X.Y., Liew, W.K., Chen, J., Chu, K.M., et al. (2011). Molecular diagnosis of severe combined immunodeficiency--identification of IL2RG, JAK3, IL7R, DCLRE1C, RAG1, and RAG2 mutations in a cohort of Chinese and Southeast Asian children. J Clin Immunol 31, 281-296.
6. den Dunnen, J.T., Dalgleish, R., Maglott, D.R., Hart, R.K., Greenblatt, M.S., McGowan-Jordan, J., Roux, A.F., Smith, T., Antonarakis, S.E., and Taschner, P.E. (2016). HGVS Recommendations for the Description of Sequence Variants: 2016 Update. Hum Mutat 37, 564-569.
7. Richards, S., Aziz, N., Bale, S., Bick, D., Das, S., Gastier-Foster, J., Grody, W.W., Hegde, M., Lyon, E., Spector, E., et al. (2015). Standards and guidelines for the interpretation of sequence variants: a joint consensus recommendation of the American College of Medical Genetics and Genomics and the Association for Molecular Pathology. Genet Med 17, 405-424.
