# Supplementary material for: Second-Tier Next Generation Sequencing Integrated in Nationwide Newborn Screening Provides Rapid Molecular Diagnostics of Severe Combined Immunodeficiency
Source: Front Immunol. 2020 Jul 9;11:1417. doi: 10.3389/fimmu.2020.01417 (PMC7381310; doi:10.3389/fimmu.2020.01417)
Supplement: Supplemental Table S6 — The individuals with the lowest TREC values on the national screening 2018-2019. [file Table_6.pdf]

Supplemental Table S6 |The individuals with the lowest TREC values in the national newborn screening program 2018 - 2019.

TRECs/µl				GA	BW	NICU	TPN	Hospital	gene panel	Known condition at birth	Findings and history	Reported and/or redraw requested	YEAR	Redraw TRECs/µl
Initial	New extraction	Re-run	Mean											
0	0	0	0	41	3540	NO	NO	Other	NBSv2	Healthy	*IL2RG*-SCID	Reported	2019	0
0	0	0	0	36	3618	NO	NO	Other	NBSv2	Healthy	Artemis-SCID	Reported	2018	0
0	0	3.4	1.1	40	2855	YES	NO	OUS	NBSv2 + PIDv2	Microcephaly, kidney anomaly	Nijmegen breakage syndrome-CID	Reported	2019	11
0	5.3	0	1.8	39	2515	NO	NO	Other	NBSv2 + PIDv2	Healthy	*JAK3*-SCID	Reported	2018	0
2.8	2.7	0.9	2.1	28	1330	YES	NO	OUS	NBSv2	Trisomy 21, hydrops fetalis, congenital heart disease, arthrogryposis	No other pathogenic variants on NBS panel	Contacted NICU-pediatrician	2019	NA
12.1	4.2	0	5.4	26	300	YES	TPN	Other	NBSv2	Extreme prematurity GA 26 <500g, Deceased 3 weeks old	No pathogenic variants detected	Redraw requested, not received	2018	NA
0	0	18	6.0	33	2220	YES	NO	Other	NBSv2	Deceased 4 days old, Congenital heart disease	No pathogenic variants detected	Not reported	2018	NA
10	2.8	5.9	6.2	34	2900	YES	TPN +BT	OUS	-	Neonatal reduced health condition Intrathoracal teratoma	No pathogenic variants detected	Redraw requested due to TPN	2019	48.0
3.2	5.1	12.1	6.8	40	3975	YES	TPN	OUS	NBSv2	Intestinal malformation	No pathogenic variants detected	Redraw requested due to TPN	2018	18.5
16.5	2.8	8.2	9.2	33	3340	YES	NO	OUS	NBSv2	Neonatal reduced health condition. Hydrops fetalis, generalized oedema. Deceased 3.5 weeks old	No pathogenic variants detected on NBS panel. Clinical WES: *PMM2*-CGD[a)]	Redraw requested	2018	30.53
22.7	2.2	7.2	10.7	32	1895	YES	TPN +BT	OUS	NBSv2	Neonatal reduced health condition, Subcapsular liver haematoma	No pathogenic variants detected	Not reported	2019	NA
3.3	4.7	24.3	10.8	29	1860	YES	NO		NBSv2	Prematurity	No pathogenic variants detected	Not reported	2018	NA
8.3	15.3	10.3	11.3	41	4065	NO	NO	Other	NBSv2 + PIDv2	Apparently healthy, and born at term with normal BW	No pathogenic variants detected, low TRECs on redraws, and few T cells on FACS, thymus aplasia on ultrasound	Redraw requested	2019	6.6-17.5
16.9	12.2	5	11.4	30	1970	YES	TPN	OUS	NBSv2	Delivery induced due to tachyarrhythmia AVRT and fetal hydrops, and ascites.	No pathogenic variants detected	Not reported	2018	NA
5.2	5.1	23.8	11.4	37	3000	YES	TPN +BT	OUS	NBSv2	Neonatal reduced health condition	No pathogenic variants detected	Redraw requested due to TPN	2018	18.8
0	25.9	8.3	11.4	38	3050	YES	NO	OUS	NBSv2	Twin	No pathogenic variants detected, and TRECs normalized	Redraw requested	2018	379.4
2.4	21.8	10	11.4	38	2870	YES	NO	Other	NBSv2	Necrotizing enterocolitis	No pathogenic variants detected	Not reported	2019	NA
12.5	4.3	17.9	11.6	39	4145	YES	NO	Other	NBSv2	Trisomy 21, congenital heart disease and intestinal malformation	No other pathogenic variants on NBS panel	Not reported	2019	NA
14.6	7.3	18.1	13.3	41	3360	NO	NO	Other	NBSv2 + PIDv2	Healthy	No pathogenic variants detected. TRECs Normalized, FACS normalized	Redraw requested	2018	159.8-281.6
8.2	15.2	18.4	13.9	31	1724	YES	TPN	OUS	NBSv2	Bilateral hydronephrosis and kidney anomaly	No pathogenic variants detected, and TRECs normalized	Redraw requested due to TPN	2019	591.3
20.2	5.8	25	17	41	3860	NO	NO	OUS	NBSv2	Born at term with normal BW	No pathogenic variants detected, and TRECs normalized	Redraw requested	2018	99.5
20.2	15	16.6	17.3	35	2185	NA	NO	Other	NBSv2	NOT KNOWN	No pathogenic variants detected	Not reported	2018	NA
8.09	31	15.8	18.3	37	2927	YES	NO	OUS	NBSv2	Congenital heart disease	No pathogenic variants detected on NBS panel	Not reported	2019	NA
20.8	28.6	6.7	18.7	41	3975	NO	NO	OUS	NBSv2	Hypospadia and tongue-tie	No pathogenic variants detected	Not reported	2019	NA

6.5	20.7	30.5	19.2	41	3366	YES	TPN	OUS	NBSv2	Congenital heart defect and microcephaly	No pathogenic variants detected on NBS panel, and aCGH 180K og MLPA DiGeorge neg.	Not reported	2019	NA
22.5	14.5	13.8	16.9	33	2190	YES	TPN	OUS	NBSv2	Prematurity GA 33, hydrothorax and arrhythmia	No pathogenic variants detected on NBS panel	Redraw requested due to TPN	2019	80.7
13	16.8	21.1	17	26	600	YES	TPN	Other	NBSv2	Extreme prematurity GA 26, <1000g	No pathogenic variants detected	Not reported	2018	NA
24.5	16.7	10.7	17.3	33	2200	YES	NO	OUS	NBSv2	Intestinal malformation	No pathogenic variants detected on NBS panel	Not reported	2019	NA
25.7	22	13.2	20.3	39	4205	NA	NO	Other	NBSv2	Born at term with normal BW	No pathogenic variants detected	Not reported	2019	NA
24.2	5	32.9	20.7	39	3075	YES	TPN	Other	NBSv2	Neonatal reduced health condition	No pathogenic variants detected	Not reported	2018	NA
21.6	17.8	24.1	21.2	40	3500	NO	NO	OUS	NBSv2	Born at term with normal BW	No pathogenic variants detected	Not reported	2019	NA
18.5	14.5	37.3	23.4	41	4590	YES	NO	Other	NBSv2	Born at term with normal BW	No pathogenic variants detected	Redraw requested	2018	238.2
20	30.02	20.4	23.5	37	3120	YES	NO	Other	NBSv2 + PIDv2	Generalized skin disease, Omenn syndrome suspected	NBS/PIDv2: homozygous VUS in *TTC7A*, but WES confirmed the diagnosis: *SPINK5* Netherton syndrome[b)]	Reported	2018	NA
17.72	12.55	41.9	24.1	40	3655	NA	NO	Other	NBSv2	Born at term with normal BW	No pathogenic variants detected	Not reported	2018	NA
22.8	18	46.7	29.2	38	3080	NO	NO	OUS	NBSv2	Born at term with normal BW	No pathogenic variants detected	Not reported	2019	NA
19.5	0	24.5	14.7	-		NO	NO	Other	NBSv2 + PIDv2	Adopted, 3 years old	No pathogenic variants detected	Not reported	2018	NA

a) NM_000303.2(PMM2):c.[357C>A];[620T>C], p.(Phe119Leu); p.(Phe207Ser)
b) NM_006846.3 (SPINK5):c.[238dup];[238dup], p.(Ala80Glyfs*19) and NM_001288951.1(TTC7A):c.[887G>C];[887G>C],p.(Ser296Thr)

Abbreviations:
aCGH, array comparative genomic hybridization/chromosomal microarray; AVRT, Atrioventricular reciprocating tachycardia**;** BT, Blood transfusion – info marked on NBS card; BW, Birth weight; CID, Combined immunodeficiency; CGD, congenital disorder of glycosylation; g, gram; FACS, fluorescence-activated cell sorting /lymphocyte flow cytometry; g, gram; GA, Gestational age – info marked on NBS card; The Human Gene Mutation Database; HGNC, The HUGO Gene Nomenclature Committee; MLPA, Multiplex ligation-dependent probe amplification; NA, Not applicable/not known; NBS, Newborn screening; NBSv2, Newborn screening gene panel version 2; NICU, Neonatal intensive care unit; OUS, Oslo University Hospital; PIDv2, Primary immunodeficiency research panel version 2; SCID, Severe combined immunodeficiency; TPN, Total parenteral nutrition – info marked on NBS card; TREC, T-cell receptor excision circles; VUS, variant of uncertain significance; WES, whole exome sequencing

Resource:
Gene names according to HGNC, https://www.genenames.org/
Gene variant nomenclature according to the HGVS recommendations[1], http://www.HGVS.org/varnomen
Gene variant classification according to ACMG standards and guidelines, Genetics in Medicine, 2015[2]

References:

1. den Dunnen, J.T., Dalgleish, R., Maglott, D.R., Hart, R.K., Greenblatt, M.S., McGowan-Jordan, J., Roux, A.F., Smith, T., Antonarakis, S.E., and Taschner, P.E. (2016). HGVS Recommendations for the Description of Sequence Variants: 2016 Update. Hum Mutat 37, 564-569.

2. Richards, S., Aziz, N., Bale, S., Bick, D., Das, S., Gastier-Foster, J., Grody, W.W., Hegde, M., Lyon, E., Spector, E., et al. (2015). Standards and guidelines for the interpretation of sequence variants: a joint consensus recommendation of the American College of Medical Genetics and Genomics and the Association for Molecular Pathology. Genet Med 17, 405-424.
