# Supplementary material for: Second-Tier Next Generation Sequencing Integrated in Nationwide Newborn Screening Provides Rapid Molecular Diagnostics of Severe Combined Immunodeficiency
Source: Front Immunol. 2020 Jul 9;11:1417. doi: 10.3389/fimmu.2020.01417 (PMC7381310; doi:10.3389/fimmu.2020.01417)
Supplement: Supplemental Table S7 — Newborn screening gene panels, versions 1-4. [file Table_7.pdf]

Supplemental Table S7 | Newborn screening gene panels, versions 1-4.

GENE NAME HGNC symbol	Disease group NBS	Core US NBS	NBS disorder, Norway 2019	NBSv1	NBSv2	NBSv3	NBSv4
ABCD1	Core US NBS genes	Core US NBS			2	3	4
ACAD8	IEM				2	3	4
ACADM	small panel IEM	Core US NBS	MCAD	1	2	3	4
ACADS	IEM				2	3	4
ACADSB	IEM				2	3	4
ACADVL	small panel IEM	Core US NBS	VLCAD	1	2	3	4
ACAT1	small panel IEM	Core US NBS	BKT	1	2	3	4
ADA	SCID and T cell defects	Core US NBS	SCID and IEM	1	2	3	4
ADK	IEM				2	3	4
AHCY	IEM				2	3	4
AK2	SCID and T cell defects		SCID/CID	1	2	3	4
ALDH7A1	IEM+				2	3	4
ALDOB	IEM+					3	4
ALPL	IEM+				2	3	4
ARG1	IEM Extended US NBS				2	3	4
ARPC1B	SCID and T cell defects		SCID/CID	1	2	3	4
ARSA	Lysosomal	Core US NBS			2	3	4
ARSB	IEM+						4
ASL	Core US NBS genes	Core US NBS			2	3	4
ASS1	Core US NBS genes	Core US NBS			2	3	4
ATM	SCID and T cell defects		SCID/CID	1	2	3	4
ATP5D = ATP5F1D	IEM+					3	4
BCKDHA	small panel IEM	Core US NBS	MSUD	1	2	3	4
BCKDHB	small panel IEM	Core US NBS	MSUD	1	2	3	4
BCKDK	IEM+				2	3	4
BCL11B	SCID and T cell defects		SCID/CID	1	2	3	4
BLNK	Antibody deficiency, low KRECs				2	3	4
BTD	small panel IEM	Core US NBS	BTD	1	2	3	4
BTK	Antibody deficiency, low KRECs				2	3	4
CBS	small panel IEM	Core US NBS	HCY	1	2	3	4
CD247/CD3Z	CID/NBS+		SCID/CID		2	3	4
CD3D	SCID and T cell defects		SCID/CID	1	2	3	4
CD3E	SCID and T cell defects		SCID/CID	1	2	3	4
CD3G	SCID and T cell defects		SCID/CID	1	2	3	4
CD40LG	Antibody deficiency, low KRECs				2	3	4
CD79A	Antibody deficiency, low KRECs				2	3	4
CD79B	Antibody deficiency, low KRECs				2	3	4
CD8A	SCID and T cell defects		SCID/CID	1	2	3	4
CDC42	CID/NBS+		SCID/CID			3	4
CDCA7	Low T cells		SCID/CID				4
CFTR	CF	Core US NBS	CF	1	2	3	4
CHD7	SCID and T cell defects		SCID/CID	1	2	3	4
CORO1A	SCID and T cell defects		SCID/CID	1	2	3	4
CPS1	IEM+				2	3	4
CPT1A	small panel IEM		CPT1	1	2	3	4

CPT2	small panel IEM		CPT2	1	2	3	4
CTNNBL1	Antibody deficiency, low KRECs					3	4
CYP11A1	small panel IEM				2	3	4
CYP17A1	small panel IEM		CAH	1	2	3	4
CYP21A2	CAH	Core US NBS	CAH		2		4
DBT	small panel IEM	Core US NBS	MSUD	1	2	3	4
DCLRE1C	SCID and T cell defects		SCID/CID	1	2	3	4
DKC1	CID				2	3	4
DLD	small panel IEM	Core US NBS	MSUD	1	2	3	4
DMD	Other				2	3	4
DNAJC12	IEM+		PKU differential			3	4
DNAJC21	SCID and T cell defects, pancytopenia		SCID/CID				4
DNMT3B	SCID and T cell defects		SCID/CID	1	2	3	4
DOCK2	SCID and T cell defects		SCID/CID	1	2	3	4
DOCK8	SCID and T cell defects		SCID/CID	1	2	3	4
EFL1 (=EFTUD1)	SCID and T cell defects		SCID/CID	1	2	3	4
EPG5	Low T cells		SCID/CID				4
ERCC6L2	Low T cells		SCID/CID				4
ETFA	small panel IEM	Core US NBS	GA2	1	2	3	4
ETFB	small panel IEM	Core US NBS	GA2	1	2	3	4
ETFDH	small panel IEM	Core US NBS	GA2	1	2	3	4
EXTL3	SCID and T cell defects		SCID/CID	1	2	3	4
FAH	small panel IEM	Core US NBS	TYR1	1	2	3	4
FOXE1 (TITF2)	Other, congenital hypothyreosis, Syndromal CH		CH			3	4
FOXN1	SCID and T cell defects		SCID/CID	1	2	3	4
G6PD	Other anemia					3	4
GAA	Core US NBS genes	Core US NBS			2	3	4
GALE	IEM Extended US NBS				2	3	4
GALK1	IEM Extended US NBS				2	3	4
GALNS	IEM+						4
GALT	Core US NBS genes	Core US NBS			2	3	4
GAMT	IEM+				2		4
GATA2	CID				2	3	4
GATM	IEM+				2	3	4
GCDH	small panel IEM	Core US NBS	GA1	1	2	3	4
GCH1	DOPA-responsive dystonia, with or without hyperphenylalaninemia		PKU differential				4
GINS1	Low T cells		SCID/CID				4
HADHA	small panel IEM		TFP/LCHAD	1	2	3	4
HADHB	small panel IEM	Core US NBS	TFP	1	2	3	4
HBB	Core US NBS genes	Core US NBS			2	3	4
HLCS	small panel IEM		HCS	1	2	3	4
HMGCL	IEM+	Core US NBS	HMGCL		2	3	4
HMGCS2	IEM+				2	3	4
HPD	IEM Extended US NBS				2	3	4
ICOSL	Low T cells		SCID/CID				4
IDS	IEM+						4
IDUA	IEM+	Core US NBS			2		4
IGHM	Antibody deficiency, low KRECs				2	3	4

IGLL1	Antibody deficiency, low KRECs				2	3	4
IKBKB	CID/NBS+		SCID/CID		2	3	4
IKBKG	CID/NBS+		SCID/CID			3	4
IKZF1	SCID and T cell defects		SCID/CID	1	2	3	4
IL21	Antibody deficiency, low KRECs						4
IL2RB	SCID and T cell defects					3	4
IL2RG	SCID and T cell defects		SCID/CID	1	2	3	4
IL7R	SCID and T cell defects		SCID/CID	1	2	3	4
IVD	small panel IEM	Core US NBS	IVA	1	2	3	4
JAK3	SCID and T cell defects		SCID/CID	1	2	3	4
KRAS	CID/NBS+		SCID/CID			3	4
LAT	SCID and T cell defects		SCID/CID	1	2	3	4
LCK	CID/NBS+		SCID/CID		2	3	4
LCRB	Core US NBS genes,	Core US NBS			2		4
LIG1	Low T cells						4
LIG4	SCID and T cell defects		SCID/CID	1	2	3	4
LMBRD1	IEM+		MMA	1	2	3	4
LRRC8A	Antibody deficiency, low KRECs				2	3	4
LYST	CID/NBS+		SCID/CID		2	3	4
MAGT1	Low T cells						4
MAP3K14	Antibody deficiency, low KRECs						4
MAT1A	IEM				2	3	4
MC2R	small panel IEM		CAH		2	3	4
MCCC1	Core US NBS genes	Core US NBS			2	3	4
MCCC2	Core US NBS genes	Core US NBS			2	3	4
MMAA	small panel IEM	Core US NBS	MMA	1	2	3	4
MMAB	small panel IEM	Core US NBS	MMA	1	2	3	4
MMACHC	small panel IEM		MMA/HCY	1	2	3	4
MMADHC	small panel IEM		MMA/HCY	1	2	3	4
MMUT	small panel IEM	Core US NBS	MMA	1	2	3	4
MTHFD1	Low T cells						4
MTHFR	IEM (large gene)				2	3	4
MTR	IEM (large gene)		HCY	1	2	3	4
MTRR	IEM (large gene)				2	3	4
NADK2	IEM				2	3	4
NAGLU	IEM+						4
NAGS	IEM+				2	3	4
NBN	CID/NBS+		SCID/CID		2	3	4
NFKB2	Antibody deficiency, low KRECs						4
NHEJ1	SCID and T cell defects		SCID/CID	1	2	3	4
NKX2-1	congenital hypothyreosis (AD), and differential CF/CID		CH			3	4
NPC1	Lysosomal				2	3	4
NPC2	Lysosomal				2	3	4
NRAS	CID/NBS+		SCID/CID			3	4
OTC	IEM+				2	3	4
PAH	small panel IEM	Core US NBS	PKU	1	2	3	4
PAX1	SCID and T cell defects		SCID/CID	1	2	3	4
PCBD1	IEM+				2	3	4
PCCA	small panel IEM	Core US NBS	PA	1	2	3	4
PCCB	small panel IEM	Core US NBS	PA	1	2	3	4

PGM3	SCID and IEM		SCID and IEM	1	2	3	4
PHGDH	IEM+				2	3	4
PIK3CD	SCID and IEM		SCID and IEM	1	2	3	4
PIK3R1	Antibody deficiency, low KRECs				2	3	4
PLPBP	IEM+						4
PNP	SCID and IEM		SCID and IEM	1	2	3	4
PNPO	IEM+						4
POR	CAH		CAH	1	2	3	4
PRKDC	CID/NBS+		SCID/CID		2	3	4
PSAP	Lysosomal				2	3	4
PSAT1	IEM+				2	3	4
PSPH	IEM+				2	3	4
PTPRC (=CD45)	CID/NBS+		SCID/CID		2	3	4
PTS	IEM+				2	3	4
QDPR	IEM+				2	3	4
RAC2	CID/NBS+		SCID/CID	1	2	3	4
RAG1	SCID and T cell defects		SCID/CID	1	2	3	4
RAG2	SCID and T cell defects		SCID/CID	1	2	3	4
RECQL4	SCID and T cell defects		SCID/CID	1	2	3	4
RLTPR (=CARMIL2)	CID/NBS+		SCID/CID		2	3	4
RMRP	SCID and T cell defects		SCID/CID	1	2	3	4
SBDS	SCID and T cell defects		SCID/CID	1	2	3	4
SLC16A1	IEM+				2	3	4
SLC22A5	small panel IEM	Core US NBS	CUD	1	2	3	4
SLC25A13	IEM				2	3	4
SLC25A15	IEM+				2	3	4
SLC25A20	small panel IEM		CACT	1	2	3	4
SLC46A1	SCID and T cell defects		SCID/CID	1	2	3	4
SLC52A2	IEM+				2	3	4
SLC52A3	IEM+				2	3	4
SLC6A8	IEM+				2	3	4
SMARCAL1	CID/NBS+		SCID/CID		2	3	4
SMN1	Core US NBS genes	Core US NBS		1	2		4
SMN2	Core US NBS genes	Core US NBS			2		4
SPINK5	SCID and T cell defects		SCID/CID		2	3	4
STAT5B	SCID and T cell defects		SCID/CID	1	2	3	4
STIM1	SCID and T cell defects		SCID/CID	1	2	3	4
TAT	IEM+				2	3	4
TBX1	SCID and T cell defects		SCID/CID	1	2	3	4
TCF3	Antibody deficiency, low KRECs				2		4
TP63	CID/NBS+		SCID/CID		2	3	4
TPP1	IEM+						4
TRNT1	Antibody deficiency, low KRECs						4
TTC7A	SCID and T cell defects		SCID/CID	1	2	3	4
UNC119	SCID and T cell defects		SCID/CID	1	2	3	4
WAS	CID/NBS+				2	3	4
ZAP70	SCID and T cell defects		SCID/CID	1	2	3	4
ZBTB24	SCID and T cell defects		SCID/CID	1	2	3	4
SLC5A5	Congenital hypothyreosis		CH			3	4
TG	Congenital hypothyreosis		CH			3	4
NKX2-5	Congenital hypothyreosis		CH			3	4
THRA	Congenital hypothyreosis		CH			3	4

PAX8	Congenital hypothyreosis		CH			3	4
TSHR	Congenital hypothyreosis		CH			3	4
DUOXA2	Congenital hypothyreosis		CH			3	4
IYD	Congenital hypothyreosis		CH			3	4
TSHB	Congenital hypothyreosis		CH			3	4
PROP1	Congenital hypothyreosis		CH			3	4
TPO	Congenital hypothyreosis		CH			3	4
SLC26A4	Congenital hypothyreosis		CH			3	4
POU1F1	Congenital hypothyreosis		CH			3	4
IGSF1	Congenital hypothyreosis		CH			3	4
CDCA8	Congenital hypothyreosis		CH			3	4
NTN1	Congenital hypothyreosis		CH			3	4
UBR1	Congenital hypothyreosis		CH			3	4
JAG1	Congenital hypothyreosis		CH			3	4
DUOX2	Congenital hypothyreosis		CH			3	4
FOXE1 (TITF2)	Congenital hypothyreosis		CH			3	4
GLIS3	Congenital hypothyreosis		CH			3	4
